# Evaluation of Primary Care Behavioral Health (PCBH) with guided self-help CBT as a treatment option – a protocol of a single-blind randomized multicenter trial (KAIROS)

**DOI:** 10.1186/s12913-025-13232-4

**Published:** 2025-09-23

**Authors:** Anneli Farnsworth von Cederwald, Sigrid Salomonsson, Nils Hentati Isacsson, Viktor Kaldo

**Affiliations:** 1 Department of Psychology, Faculty of Health and Life Sciences, Linnaeus University, Växjö, Sweden; 2 Centre for Psychiatry Research, Department of Clinical Neuroscience, Karolinska Institutet, & Stockholm Health Care Services, Region Stockholm, Stockholm, Sweden

**Keywords:** Primary care behavioral health, PCBH, Guided self-help, GSH, Cognitive behavioral therapy, CBT, Primary care

## Abstract

**Background:**

While protocol-based psychological treatments have significantly advanced mental health care, real-world accessibility remains a challenge. Primary care, the main provider of mental health services, faces barriers such as limited resources and a diverse patient population with varying needs, making it difficult to rely solely on time-intensive, protocolized treatments. The Primary Care Behavioral Health (PCBH) model promotes brief, flexible interventions that may better accommodate these needs. However, limited research on these interventions raises concerns about potential undertreatment. To align with Universal Health Coverage principles, it is essential to identify which patient groups benefit most from resource-efficient protocol-based versus brief, flexible, and individualized treatments. Our main aim is to evaluate whether a integrating guided self-help into PCBH improves outcomes compared to the core PCBH model, as well as to assess whether patients identified as suitable for protocol-based interventions benefit more from the combined model.

**Methods:**

Patients seeking help for mental or behavioral health problems at PCBH primary care centers will be randomized to one of two arms: core PCBH, where patients receive a contextual assessment and brief interventions tailored to their needs, or an extended PCBH model, where a diagnostic assessment determines whether patients receive brief interventions or guided self-help. The primary outcome is functional impairment, assessed at baseline and followed up at 4, 8, and 12 weeks (primary endpoint), as well as at 1 year. Secondary outcomes include symptom change, cost-effectiveness, and care process factors.

**Discussion:**

The study design allows for comparisons of patient outcomes between the two care models, with a primary focus on evaluating superiority and a secondary focus on non-inferiority, cost-effectiveness, and care process factors. Overall, the project seeks to advance understanding of effective mental health interventions in primary care settings and inform decision-making regarding treatment approaches.

**Trial registration:**

ClinicalTrials.gov: NCT04900064. Registered on May 25th, 2021. Registered with the Swedish Ethical Review Board (2020–04198) on October 12th, 2020. This protocol was submitted for publication on March 18th, 2025, prior to the inclusion of the final participant, and will shortly thereafter, without any changes, be made publicly available as a preprint in an open-access repository.

**Supplementary Information:**

The online version contains supplementary material available at 10.1186/s12913-025-13232-4.

## Background

Over the past decades, the development of empirically supported psychological treatments (ESTs) has been remarkable [1]. Most ESTs are protocol-based cognitive behavioral therapies (CBT) tailored to specific psychiatric disorders [1]. A substantial body of research shows that these can be used to effectively treat common mental health conditions such as depression [2, 3], anxiety disorders [4–8], and insomnia [9]. Despite this progress, the prevalence of mental health issues has not declined at the population level [10], suggesting a gap between advances in research on ESTs and their real-world impact.

Primary care serves as the first point of contact within the healthcare system, ideally providing accessible, person-centered care for a wide range of health concerns. It plays a central role in achieving Universal Health Coverage, which emphasizes equity, access, quality, and financial protection. Given the burden of mental health conditions, it is crucial to understand how psychological interventions can be effectively integrated into primary care to improve public health. However, significant challenges remain in translating the current ESTs into primary care settings. ESTs are often designed for specialist care settings, where treatment intensity and patient selection differ from the realities of primary care. One major issue is limited access: in Sweden, for example, only about 3% of primary care patients have any contact with a mental health professional [11], despite estimates suggesting a point prevalence of depression and anxiety of 17.2% [12]. In fact, ESTs may face even greater scalability challenges – Strosahl and Robinson [13] estimate that a typical primary care clinic would need 12 full-time therapists just to provide in-person ESTs to all patients with depression, underscoring the gap between theoretical efficacy and practical feasibility.

Another key challenge is treatment uptake – although approximately 50% of patients respond to ESTs, this proportion drops to around 20% when accounting for ineligibility, non-attendance, and dropout rates [14]. A possible explanation for these challenges is that solely relying on protocol-driven ESTs may not be well-suited to the primary care context. Primary care patients seek help not only for diagnosable psychiatric disorders but also for broader concerns such as grief, relationship problems, and stress related to work or family issues. Structured assessment tools and diagnostic interviews, while valuable for ensuring accurate diagnosis and guiding appropriate treatment decisions, may inadvertently lead some patients to feel that their concerns are not adequately addressed. This may lead to patients discontinuing treatment prematurely [15].

To address these challenges, population-based healthcare models such as Primary Care Behavioral Health (PCBH) [17] emphasize brief, adaptable interventions that can reach a broader patient population. These approaches often extract core treatment strategies from EST protocols, making them more flexible and responsive to individual patient needs. In many ways, these Brief Interventions (BI) echo the sentiments of process-based therapy, where treatment decisions are based on an assessment of underlying psychological processes rather than diagnoses [16]. However, such adaptations also raise concerns: without systematic assessments followed by well-supported ESTs in their full format, some patients who could benefit from protocol-based treatments may receive suboptimal care.

A key question is how primary care can balance the need for flexible, scalable interventions with the presumed benefits of structured, evidence-based treatment protocols for those who need it. One possibility is to tailor assessment and interventions based on patient needs and circumstances. Patients with subclinical symptoms, challenges primarily related to life stressors, or practical and economic barriers to engaging in time-intensive treatment may benefit more from brief, individualized interventions. In contrast, those with identified psychiatric disorders who can actively participate in structured treatment might achieve better outcomes with protocol-based interventions. Empirical evidence also suggests that some disorders, such as obsessive-compulsive disorder (OCD), require highly specific interventions to achieve meaningful improvement [18], whereas others, such as depression, seem to respond to a wide range of treatment approaches [2]. Identifying which patient groups benefit most from structured ESTs versus flexible, brief interventions could help optimize both access and treatment effectiveness, aligning primary care mental health services with Universal Health Coverage principles.

### Primary Care Behavioral Health (PCBH) and brief interventions (BI)

PCBH emphasizes delivery of brief and acceptable interventions to all patients as part of clinic routine, promoting same-day availability and high productivity [17]. Psychologists or counselors, known in the model as Behavior Health Consultants (BHCs), are core members of the primary care team, working closely with physicians, nurses, and other health professionals in addressing both mental health and behavior-related physical ailments. The model relies on teamwork and task-shifting to ensure cohesiveness for patients and reduce service bottlenecks. While many therapists working within the PCBH framework utilize CBT interventions [18], the delivery model is significantly different. Unlike many ESTs, which follow a structured timeline with a predetermined endpoint (e.g., one session per week for 12 weeks), the PCBH model operates on the presumption that health-promoting behaviors, skills, and habits require ongoing reinforcement – potentially over a lifetime [13]. As such, each individual episode of care is brief, both to ensure they are accessible to the entire population and to allow patients the opportunity to return as needed. Patients typically have 30-minute visits scheduled one at a time. While follow-ups can be booked as needed to assess progress and adjust treatment, the model encourages patients to book additional visits when the need arises rather than pre-planning extended treatments. The assessment procedure, described in detail below, is less focused on diagnostics and more concerned with making an individual conceptualization of how the patient’s context and current coping strategies are connected to their symptoms. An evidence-informed approach is utilized, where modularized core concepts, commonly inspired by the ESTs, are chosen based on an individual analysis [13]. These treatments are commonly referred to as brief interventions (BI).

The practices of PCBH may help expand access to and uptake of psychological interventions [19, 20]. On the other hand, previous studies on the model have limited scientific validity due to consistent methodological weaknesses, where a large problem is the absence of controlled designs. Available pre-post studies suggest that BI in the PCBH framework may improve symptoms, functioning, and well-being across various problem areas, but there is a significant need for higher-quality research [21, 22].

### Guided self-help

Guided self-help (GSH) involves structured, disorder-specific treatments – often grounded in established CBT protocols – delivered through resources such as books or online programs, typically accompanied by limited clinician support [23, 24]. Extensive evaluations of GSH have demonstrated its effectiveness in alleviating symptoms of anxiety and depression, with outcomes comparable to face-to-face CBT [25, 26], while using approximately half the resources [24]. In a primary care setting, research specifically on illness anxiety disorder has shown that self-help books have shown similar effectiveness to internet-based treatments and yielded superior effects compared to waitlist controls [27]. A primary care study of stepped care for common mental disorders found that using self-help books with limited therapist guidance as a first step led to remission in 40% of patients [24].

In summary, GSH appears to be a more resource-efficient alternative to face-to-face CBT while delivering comparable outcomes. However, these treatments remain best suited for patients whose primary or significant concerns align well with existing treatment protocols and who are able and willing to engage in them. In contrast, those whose needs fall outside these parameters may benefit more from BI used in PCBH. This raises an important question: can a resource-efficient version of protocol-driven treatments such as GSH be effectively integrated with high-uptake, individually adaptable interventions within a PCBH-based care model?

### Aim and purpose

The main purpose of the project is to evaluate if adding GSH as a treatment option within the PCBH framework (PCBH + GSH) – offered only to patients deemed suitable following a brief, structured assessment – can enhance treatment outcomes compared to the core PCBH model. Patient outcomes in the research questions below include everyday functioning (primary outcome) as well as disorder-specific symptoms. The research questions are as follows:

1. Primary question: When comparing patient outcomes at the care model level after 12 weeks, is PCBH + GSH more effective than the core PCBH model? If not, can non-inferiority of core PCBH be established?
2. Among the subgroup of patients deemed suitable for structured interventions in both groups, do those receiving GSH in PCBH + GSH achieve better outcomes than those receiving BI in core PCBH? Additionally, is this effect more pronounced in patients with conditions expected to require specific treatment components, such as exposure therapy for anxiety disorders, compared to those with conditions like depression and stress, where the benefits of specific treatment components are less clearly established? If not, can non-inferiority of BI be established for these subgroups?
3. Does adding GSH to PCBH increase treatment duration or costs of care delivery? Which model is more cost-effective, considering patient benefits in relation to healthcare and societal costs?
4. Do the care models and treatments differ in patient satisfaction, adverse events, treatment content, and which behavioral changes patients make during and after treatment?
5. Will the protocol-based intervention of GSH decrease variation in treatment outcome compared to BI, especially when comparing patients deemed suitable for structured interventions?
6. In BI, do patients whose therapists use techniques aligned with evidence-based strategies for the patient’s specific symptoms (e.g., exposure for anxiety) achieve better outcomes? How do outcomes differ for various subgroups of patients receiving BI under different conditions, and how do these compare to the group receiving GSH? In GSH, how do therapists’ adherence to treatment protocols impact outcomes? More broadly, how does the specific treatment content, independent of which type of intervention it is provided in, influence patient outcomes?

Additionally, we want to explore if the PCBH + GSH model is feasible for large-scale implementation in healthcare.

7. What proportion of patients are considered suitable for GSH following an extended diagnostic assessment? How does this proportion vary across different primary care centers and over time within a center? What factors contribute to this variation?
8. How do patient-related outcomes differ between centers and therapists, and what factors contribute to these variations? Do these differ depending on which treatment is given?
9. To what extent are GSH treatments delivered with fidelity to GSH principles within the PCBH framework, particularly in areas where PCBH and GSH principles conflict (i.e. booking practices, prioritizing treatment fidelity versus maximizing access)? Are BI treatments delivered in accordance with PCBH principles and the evidence-base? How does fidelity vary across therapists and primary care centers, and what factors contribute to this variation?

## Methods/design

### Preregistrations and current protocol

The study was preregistered on May 25th, 2021, prior to the inclusion of the first patient (ClinicalTrials.gov: NCT04900064). Before this, the study was outlined in an ethical review application to the Swedish Ethical Review Board (2020–04198) on October 12th, 2020. This is the first version of the study protocol (1.0), developed toward the conclusion of the data collection phase. It was submitted for publication on March 18th, 2025, prior to the inclusion of the final participant, and will shortly thereafter, without any changes, be made publicly available as a preprint in an open-access repository. The protocol serves to provide detailed specifications on the study methods and analyses before analyses start. It has been developed in accordance with the SPIRIT guidelines [28].

While this protocol, the preregistration and the ethical review application largely align, some differences exist. Due to challenges in performing the coding practice for which patients in the core PCBH arm would be suitable for structured interventions, identified in a pilot study to this trial, the comparison based on that coding is now classified as secondary instead of primary. Additionally, some implementation outcomes outlined in the preregistration – such as whether the implementation of GSH increases waiting times – will be excluded due to inherent limitations of the study design, where the presence of two parallel care models within the same center renders any results from these outcomes non-informative. Research questions regarding variations in what proportion of patients are assessed as suitable for GSH, adherence to GSH principles, between-center variation and therapist effects (questions 5–9 above) have been added, following observations during clinical supervision throughout the trial. Trial reports will include a dedicated section for reporting deviations.

### Design and overall procedure

The proposed study is a single-blind, multicenter, randomized clinical trial with a 1:1 allocation ratio at the individual level. It will feature two study arms and four measurement points. Potential participants will be identified prior to their first visit. For those who provide written consent, the pre-treatment assessment will be conducted either shortly before or within seven days of their initial visit. This flexibility is necessary to accommodate the typically very brief waiting period between patients’ self-referrals and their first visit. Follow-up assessments will be conducted at 4, 8, and 12 weeks, with the 12-week point serving as the primary endpoint. Standard Operating Procedures (SOPs) will be implemented to ensure consistency in key procedures, and digital Client Report Files (CRFs) will be maintained on the study’s technical platform. Participants will be randomly assigned to one of two study arms:

1. Core PCBH model (PCBH): All patients undergo a contextually based interview (described below) to assess their concerns, symptoms, context and values, and are then offered BI. Retrospectively, patients are assessed to determine whether they would have been indicated for a structured, protocol-based treatment. However, this does not affect which intervention they receive, as all patients are given BI.
2. Extended PCBH model (PCBH + GSH): PCBH practices are complemented with an extended assessment, including a brief, standardized diagnostic procedure to determine if GSH is a suitable treatment option for the patient. Patients that are deemed suitable for and accept GSH as an intervention receive a condition-specific self-help manual along with clinician guidance; those not suitable receive BI.

This means that patients are not randomized between treatments, but between models of care (PCBH vs. PCBH + GSH). In the extended arm (PCBH + GSH), patients will receive either GSH or BI based on their assessed suitability for GSH treatment. Suitability in this context means that the patient is both interested in and capable of engaging in a self-help treatment and has a primary problem related to mood, anxiety, stress-related conditions, or insomnia. In contrast, the core PCBH arm will include only patients who have received BI. However, within this group, there will be patients would have been suitable for GSH if it was available to them. After data collection is complete, these patients will be retrospectively identified using a standardized procedure that as closely as possible mirrors the extended assessment in the PCBH + GSH model. This approach will allow us to address the secondary research question [2] regarding the subgroup of patients suitable for GSH. The retrospective assessment can determine whether the patient’s primary problem falls within the specified categories and identify any clear indications that they would not be capable of engaging in treatment. However, patient preferences remain unknown, as they were never offered the option. Refer to Table 1 for an overview of the patient groups, along with the codes used to identify them.

**Table 1:** Patient groups by randomization, indication for structured interventions, and treatment given

Model of care	Indicated for a structured intervention	Treatment received	Code
**Core PCBH**	Indicated	BI	CORE-I-BI^1^
	Not indicated	BI	CORE-N-BI^2^
**Extended PCBH**	Indicated	GSH	EXT-I-GSH^3^
	Not indicated	BI	EXT–N-BI^4^

^1^Retrospectively indicated for a structured intervention for mood, anxiety, stress-related conditions, or insomnia but received brief interventions (BI)

^2^Retrospectively not indicated for a structured intervention and received brief interventions (BI)

^3^ Assessed by the clinician as indicated for a structured intervention and received guided self-help (GSH)

^4^ Assessed by the clinician as not indicated for a structured intervention and received brief interventions (BI)

Note: In the Core PCBH arm, indication for a structured intervention is assessed retrospectively by an expert rater. In the Extended PCBH arm, indication is assessed in real-time by the regular care clinician

### Enrollment of PCBH PCCs

PCCs that have already implemented and consistently adhere to the PCBH model are eligible for inclusion in the trial. The PCCs in this study adhere to a PCBH model adapted to the Swedish healthcare system [29]. To ensure adherence, a qualitative evaluation of PCBH fidelity will be conducted at each participating PCC by one of the authors (AFvC) prior to trial inclusion. This evaluation will involve assessing key aspects of PCBH implementation, including the integration of at least one BHC who is skilled in delivering BI. A list of study sites is available at the Clinical Trials preregistration.

### Participant recruitment

Trained research assistants will gain access to the PCC’s booking systems and will twice a day scan the schedules of BHCs to identify, contact and randomize potential participants. All patients aged 18 and older who are deemed suitable for mental or behavioral health interventions – as assessed by nurses or other PCC staff as part of standard routines – will be provided with information about the study. This applies regardless of their stated reasons for seeking healthcare, potential diagnoses, or concurrent medical treatments. The inclusive eligibility criteria align with the naturalistic setting and aim of the PCBH model. However, patients who are already undergoing psychotherapeutic treatment or have visited BHCs at the PCC for the same reason within the past three months will not be eligible for participation. Patients will primarily be contacted by phone, with text messages used as an alternative if they are difficult to reach. Contact attempts will begin as soon as patients are identified and will continue until their first visit has passed. All identified patients will receive digital access to written information about the study, consent forms, and pre-measure screening tools via the phone call or text message.

### Randomization and blinding

The independent Karolinska Trial Alliance (KTA) has set up a randomization system on the website randomize.net, based on list randomization with even blocks of random sizes determined by KTA and not shared with the researchers. Research assistants, who are not involved in other aspects of the trial and have limited knowledge of the differences between the study arms, will conduct each individual randomization using this website. The platform ensures concealment and integrity by performing real-time allocation, revealing the treatment assignment only after participant enrollment, and maintaining a comprehensive audit trail with timestamps and user activity logs. To minimize placebo and nocebo effects, participants will remain blind to the availability of different treatment options in the study arms. They will be briefly informed that the PCC is evaluating two types of assessments, both more comprehensive than standard routine. As the PCBH arm also includes extensive pre-treatment questionnaires, which are not part of standard PCBH procedures at the PCCs, this design ensures that both study arms are perceived by participants as offering a more thorough assessment. However, participants will not be told which assessment they were allocated to, ensuring blinding to both the type of assessment and treatment options.

Given the prompt availability of PCBH PCCs, often providing same-day appointments, it is worth noting that participants in this study are randomized immediately upon identification, even before completing the pre-measure. The randomization result is noted in the clinician’s schedule, allowing them to conduct assessments in accordance with the randomization during the initial visit. However, only patients who consent to the study and fill in the pre-measure will be included in analyses.

### Models of clinical assessment

#### Contextual assessment (Core PCBH arm)

Clinicians in PCBH place a significant importance on understanding the patient’s current context and their strategies for managing stressors and unwanted experiences. The therapist explores the patient’s life context using a semi-structured interview called the Contextual Interview (CI), focusing on finding what triggers the unwanted experiences, when and how often they occur, and the trajectory of the problem. The goal is to promote a person-centered, whole-person perspective, while collecting clinically relevant information (such as avoidance behaviors, life skill deficits and dysfunctional coping strategies) to facilitate individually tailored interventions. In this model, the CI is regarded as a therapeutic intervention in its own right as opposed to a way of merely collecting information, as it broadens the patient’s perspective, helping them see their symptoms and problems within a wider context [29]. This shift in perspective is thought to foster insights that may inspire new ideas or behaviors. As such, a collaborative effort to identify and introduce an intervention is made during the first visit, directly after the CI. While the initial assessment and first intervention are typically completed within a single half-hour visit, both the assessment process and intervention strategies can be further refined during subsequent patient visits if needed. In contrast to the diagnostic assessment described below, the clinician pays little attention to whether the patient fulfils the criteria for a psychiatric diagnosis. In this study, clinicians do however have access the answers of the pre-treatment questionnaires, including the result of the automated diagnostic screening algorithm, and choose freely if they use them. As the study is integrated into routine healthcare, patients requiring more specialized care will be referred to an appropriate level of care irrespective of their randomization assignment.

#### Extended diagnostic assessment (Extended PCBH arm)

Manualized CBT operates within the medical model of psychiatry, which means that the aim of treatment is to achieve remission of an identified disorder or condition [1]. It is therefore seen as pertinent to assess which diagnosis a patient suffers from, so that the correct protocol can be chosen. The demands for a structured assessment are even greater in GSH, as the standardized self-help material limits the possibility for re-assessment, adaptions and individualization of content during treatment.

In this study, the diagnostic assessment consists of three parts: an automated algorithm described below (see Measures), based on patients’ pre-treatment self-ratings to screen for diagnoses and overall suitability for GSH, an interview focused on the patient’s medical history, symptoms and life context, and the use of selected parts of Mini-International Neuropsychiatric Interview (M.I.N.I.) [30] based on which problem areas the algorithm identified as relevant. The assessment takes place over two 30-minute visits. A key difference between the two types of assessment is that, in the diagnostic assessment, there is a clear distinction between assessment and intervention. The assessment is completed first, and only after that is a decision made about which interventions to proceed with at a subsequent visit. While some questions may naturally overlap (such as those regarding the life context), in this form of assessment, they are primarily seen as a means of gathering information rather than initiating an intervention.

The clinician is instructed to always use the results of the automated algorithm to inform the focus of the M.I.N.I. interview. As the GSH treatments in this study are possible to use with subclinical problems, the goal of the assessment is not necessarily for the patient to meet a specific diagnosis but rather to pinpoint the problem area most relevant to the patient’s concerns. The diagnostic evaluation concludes with the clinician providing feedback to the patient regarding the assessment results and, when appropriate, offering GSH for the identified condition. If GSH is not a good fit or the patient declines the treatment, they are offered BI. In such cases, BI may be initiated during the first visit if time permits or is otherwise scheduled for a follow-up appointment. Clinicians may also bypass the diagnostic assessment and proceed directly with BI if it is clear from the outset that the patient is unsuitable for GSH. Examples of this are if the patient’s primary concern clearly falls outside the issues addressed by the GSH materials used in this study (for example substance abuse). As in the contextual assessment, patients requiring specialized care will be referred accordingly.

### Interventions

#### Brief interventions (BI)

Typically spanning from a single to a few sessions, BI are customized to meet the immediate needs and objectives of the patient. Based in the contextual assessment, clinicians collaborate with patients to conceptualize the problem as well as establish a plan with achievable goals. Psychoeducation, reframing, normalization, and validation often play important roles in these interventions, providing patients with an understanding of how their problems interact with their inner and outer context. Additionally, BI often incorporate skill-building techniques. Common clinical practice include using chosen parts of ESTs (for example, working with behavioral activation or exposure principles, but not following the recommended session length or amount), or using treatment options designed to be brief, such as Focused Acceptance and Commitment Therapy (FACT), which emphasizes mindfulness, acceptance, and value-based action to help individuals develop psychological flexibility [31]. Following the initial intervention, clinicians may schedule follow-up appointments or check-ins to evaluate progress, reinforce learning, and make any necessary adjustments to the treatment plan.

#### Guided self-help (GSH)

In this study, GSH is administered through self-help books tailored to specific disorders and supplemented with both individual 30-minute guiding sessions (conducted in-person or via video) and shorter 15-minute follow-ups (conducted over the phone or through brief video calls). Each GSH treatment contains between 3 and 6 contacts, of which a minimum of 1 and a maximum of 3 are longer guidance sessions. In contrast to BI practices, all guiding sessions are pre-planned at the outset of treatment. These are scheduled at key points in treatment, such as when the patient is starting exposure exercises in GSH targeting anxiety. The brief check-ins do not have to be pre-planned but can be incorporated as needed throughout the treatment, allowing for increased frequency for patients who require additional support. The role of the therapist is to motivate the patient to follow the program, especially during more demanding parts of the treatment, and to actively engage in practicing challenging exercises together with the patient. Each chapter of the books corresponds to the content of one session of face-to-face CBT, featuring psychoeducation, and practical exercises for documenting and challenging thoughts, emotions, and behavioral patterns contributing to the disorder. Typically, patients progress at a pace of one chapter per week, though this tempo can be adjusted based on individual needs.

GSH is offered using CBT self-help books for depression [32], generalized anxiety and worry [33, 34], illness anxiety disorder [35], social phobia [36], panic disorder [37], OCD [38], stress, adjustment disorder and exhaustion disorder [39], and insomnia [40]. All used books have been previously evaluated in research to ensure their efficacy and suitability, either in book format or text-based internet treatments [24, 32, 34, 41–46]. For depression and worry, the project has self-printed books based on internet treatments. For the depression book, some modifications were made to the original text, incorporating a stronger values-based component in behavioral activation, rather than focusing solely on increasing positive reinforcement [47]. The generalized anxiety and worry book underwent only minor changes to improve its appearance in print, with the content largely unchanged [34].

#### Training and supervision

As noted above, only PCCs with high fidelity to the PCBH model are selected for the study, aiming to ensure that clinicians are proficient in delivering BI. Recently hired clinicians with limited experience in PCBH or BI are provided with six half-day training sessions. Additionally, all participating clinicians receive at least monthly supervision in BI as part of their routine practice. However, diagnostic assessments and GSH are expected be new to the clinicians in PCCs operating under the PCBH model. As such, all clinicians undergo a 4-hour online training covering diagnostic assessment and general principles for GSH. They receive copies of all books used within the GSH treatment with reading instructions, as well as recorded lectures on the books and underlying evidence. Additionally, every book comes with a brief clinician’s guide, delineating important treatment components and when guidance sessions should be booked. Throughout the study duration, clinicians administering GSH to patients receive 45-minute clinical supervision sessions every two weeks in addition to any supervision they have in their clinic.

### Measures

#### Sociodemographic and clinical data at baseline

Self-reports will be used to gather information on relationship status, education, occupation, financial stability, emotional and practical support, and living arrangements. Additionally, self-reports and medical records will be utilized to document the main reason for the visit, problem duration, other health concerns, concurrent care, ICD-10 diagnoses, and medications. These forms are available in the Supplementary materials A.

#### Patient outcomes

Patient outcomes will be primarily assessed through self-report forms, complemented by a semi-structured interview to gather more detailed information about the patient experience. Self-report measures are completed at the pre-measure timepoint – prior to or shortly after the initial visit at the PCC – with follow-ups at 4 weeks (FU4), 8 weeks (FU8), and 12 weeks (FU12), with FU12 serving as the primary endpoint. A long-term follow-up is conducted one year later (FU52). The number of visits and contacts during this period is variable in both BI and GSH treatments. All timepoints are relative to the completion of the pre-measure. The interview, available in the Supplementary materials B, is conducted at the FU12 timepoint and allows patients to provide detailed feedback on their satisfaction, adverse events, treatment content, goals, and perceived changes in symptoms and quality of life.

##### Everyday functioning – Primary outcome

The primary patient outcome is everyday functioning, assessed through 8 out of 12 items covering four domains of the WHO Disability Assessment Schedule 2.0 (WHODAS-12) [48]: Life activities, Cognition, Getting along, and Participation, together reflecting psychosocial functioning. The Mobility and Self-care domains, more representative of physical functioning, have been found to constitute independent factors in mental health patients [49] and are expected to remain relatively stable throughout treatment. Hence, they will be excluded in primary analyses, but will be included in secondary analyses for better comparability with other trials.

##### Symptoms

Abbreviated versions of established patient-rated questionnaires for prevalent mental health disorders will be used to measure symptoms. See Table 2 for an overview, including timepoints and cut-off scores. Each scale score will be normalized to a range of 0 to 1 by dividing it by the scale’s maximum value. This normalized value will be used in two ways:

1. Primary symptom: Patients indicated as suitable for structured treatments (the CORE-I-BI and EXT-I-GSH groups) will have their primary symptom measure determined based on which GSH intervention is deemed most suitable for them. The stress and exhaustion areas, which are closely related and treated using the same GSH protocol, are assessed using two separate scales. The primary symptom measure is the averaged value of both scales, though they will also be analyzed separately. Patients indicated for BI (the CORE-N-BI and EXT-N-BI groups) will not have a value for this measure.
2. General symptom index: A composite measure will be calculated by averaging the normalized scores across all symptom scales at each measurement point. To prevent the similar scales of stress and exhaustion from disproportionately influencing the overall measure, their average will be used as a single combined score in the general symptom index. AUDIT-C and the pain scale will be excluded, as substance use and pain are not targeted by the interventions.

**Table 2:** Overview of patient questionnaires including scale origin, algorithm cut-offs, and administration timepoints

Self-report questionnaires	Origin	Algorithm cut-off	PRE	FU4	FU8	FU12	FU52
*Functioning*,* quality of life and health behaviors*							
Brunnsviken Brief Quality of Life Scale 12-items (BBQ)	PUB		X			X	X
DAily Routines for Well-being INventory 13-items (DARWIN)	CON		X			X	X
Outcome Rating Scale 4-items (ORS)	PUB		X	X	X	X	X
World Health Organization Disability Assessment Schedule 2.0 12-items (WHODAS 2.0)	PUB		X			X	X
**(Primary Outcome) World Health Organization Disability Assessment Schedule 2.0 8-items (WHODAS 2.0)**	**PUB**		**X**	**X**	**X**	**X**	**X**
*Symptoms*							
Alcohol Use Disorders Identification Test-Concise (AUDIT-C-3)	PUB	3–4^1^	X	X	X	X	X
Generalized Anxiety Disorder 2-item (GAD-2)	PUB		X	X	X	X	X
Generalized Anxiety Disorder 7-item (GAD-7)	PUB	9	X			X	X
Insomnia Severity Index 2-item (ISI-2)	PUB	4	X	X	X	X	X
Karolinska Exhaustion Disorder Scale 3-item (KEDS-3)	CON	11^2^	X	X	X	X	X
Obsessive Compulsive Disorder 3-item (OCD-3)	CON	3	X	X	X	X	X
Pain (One-item rating from 0 to 10)	CON		X	X	X	X	X
Panic Disorder Severity Scale 2-item (PDSS-SR-2)	EMP	2	X	X	X	X	X
Patient Health Questionnaire 2-item (PHQ-2)	PUB		X	X	X	X	X
Patient Health Questionnaire 9-item (PHQ-9)	PUB	8	X			X	X
Perceived Stress Scale 3-item (PSS-3)	CON	8	X	X	X	X	X
Short Health Anxiety Inventory 3-item (SHAI-3)	EMP	3	X	X	X	X	X
Social Phobia Inventory – Abbreviated Version (Mini-SPIN-3)	PUB	5	X	X	X	X	X
*Questionnaires used for health economic analyses*							
EuroQol-5 Dimensions (Eq. 5D)	PUB		X			X	X
Trimbos/iMTA questionnaire for Costs associated with Psychiatric illness (TiC-P)	PUB		X			X	X
Question about sick leave 1-item				X	X		
*Treatment credibility*,* satisfaction and adverse events*							
Adverse events 3-item	CON			X	X		
Adverse events 9-item	CON					X	
Assessment of goal achievement (Evaluation of change) 2-items	CON					X	
Assessment of your mental health care at the PCC 9-items	CON					X	
Client Satisfaction Questionnaire (CSQ) 4-items	CON					X	
Patient Global Impression Improvement 1-item (PGI-I)	PUB			X	X	X	X
Session Rating Scale 5-items (SRS)	PUB			X	X	X	
Treatment Credibility Scale 5-items	PUB		X				
*Sociodemographic and clinical data*							
Background questions 10-items			X				
Questions about care outside the study 4-items						X	
Questions about health issues and care 6-items			X				

^1^ The cut-off is set to 4 for men and 3 for women as well as patients with other gender identities

^2^ To reach the cut-off, it is also required that the patient attributes their symptoms to long-term stress

Note: The origin of the measure is defined as follows: PUB (published and empirically tested); EMP (created empirically through factor analytic item-reduction and sensitivity-to-change analyses on large datasets from previous trials conducted by the research group, not yet published); CON (created through consensus by the researchers or an expert group, not yet published or empirically tested)

The symptom scales are also used by the automated algorithm to help identify patients’ main areas of concern. If a patient scores above the cut-off specified in Table 2, points will be added to an overall score reflecting the relevance of the problem area for the patient. This process is further detailed below. The cut-offs, set by the research group, are not intended to indicate likely diagnoses but to highlight problem areas that may be significant for the patient. When available, established cut-offs have been used –however, these have been adjusted for the purpose of this study when necessary, for example to achieve a better balance between the problem areas. In cases where no established cut-offs exist, the research group has reached a consensus to define appropriate thresholds. This tailored approach enhances the algorithm’s ability to identify clinically meaningful issues while minimizing false positives for common experiences. The symptom scales, their cutoffs and their use in the algorithm were tested in a pilot study (manuscript in preparation). However, three scales measuring symptoms of stress, exhaustion disorder and OCD were adjusted or replaced based on clinical observations during the pilot study. The modifications aimed to enhance clinical relevance and ensure that the scales more accurately capture the core symptoms of each condition.

The PHQ-9 is a self-report tool used to assess the severity of depression symptoms over the past two weeks [50]. It consists of nine questions covering common symptoms of depression, such as low mood, loss of interest or pleasure, and changes in appetite or sleep patterns. The GAD-7 is a questionnaire designed to measure the severity of generalized anxiety disorder symptoms [51]. It comprises seven items assessing symptoms such as nervousness, worrying, and restlessness over the past two weeks. PHQ-2 and GAD-2 are concise versions incorporating two items from each of these scales, assessing depression and anxiety symptoms [52, 53], together forming the PHQ-4 scale [54]. The PDSS-SR-2 is a two-item scale used to assess the severity of panic disorder symptoms, measuring the frequency and severity of panic attacks over the past week [55]. Mini-SPIN is a 3-item questionnaire used to assess symptoms of generalized social anxiety disorder, evaluating fear, avoidance, and physiological symptoms related to social situations [56]. ISI-2 is a shortened version of the Insomnia Severity Index used to assess the severity of insomnia symptoms [57]. It consists of two items evaluating the severity of difficulty falling and staying asleep. AUDIT-C is a brief screening tool used to assess alcohol consumption patterns and identify individuals at risk for alcohol-related problems [58]. It consists of three questions assessing alcohol consumption, frequency of binge drinking, and alcohol-related problems. Bodily pain was measured with a 10-point Likert scale, where 0 indicates no pain over the past two weeks and 10 represents the worst pain imaginable during the same period.

For illness anxiety disorder, stress, exhaustion disorder, OCD and pain, there is a lack of validated ultra-brief scales. In these cases, scales have been developed through one of three approaches: (1) factor analytic item-reduction and sensitivity-to-change analyses on large datasets from unpublished studies conducted by the research group (2), expert consensus to select items from longer validated scales, or (3) expert consensus to create entirely new brief scales. The SHAI-3 contains 3 items [5, 7, 10] from the SHAI-14 [59], measuring symptoms of illness anxiety disorder, selected through factor analysis. To measure stress and exhaustion, three items [3, 8, 9] were selected from the Perceived Stress Scale-14 [60] and three [1, 4, 5] from the 9-item KEDS [61], which assesses symptoms of exhaustion disorder, forming the PSS-3 and KEDS-3 instruments, respectively. These items were selected by the research group in collaboration with a leading expert in Swedish stress research to capture the patient’s intuitive experience of stress, while minimizing overlap with closely related disorders. A brief measure used to assess symptoms of obsessive-compulsive disorder was developed together with a leading Swedish OCD researcher. The measure consists of a description of obsessive thoughts and compulsions with examples of common themes and behaviors, asking if the patient has had any obsessive thoughts or compulsive behaviors and if so, how uncomfortable or difficult the experience has been. The OCD scale is available in Supplementary materials C.

##### Quality of life and self-care

Secondary measures include the 12-item Brunnsviken Brief Quality of Life (BBQ) questionnaire, which evaluates the importance and fulfillment of six areas (e.g., spare time, creative work, and friendship) [62]. Additionally, the 4-item Outcome Rating Scale (ORS) [63] will be used. Complementing these measures is the DAily Routines for Well-being INventory (DARWIN), developed by the research group (see Supplementary materials C), which evaluates the frequency of 11 behaviors relevant to mental health, including exercise, regular eating, socializing, and sleep hygiene.

##### Patient experiences, satisfaction, adverse events and treatment credibility

Patient experiences and perceptions will be measured using self-report forms, as well as further explored qualitatively during the FU12 interview. Subjective change in the patient’s condition since seeking care will be assessed using the one-item Patient Global Impression - Improvement (PGI-I) scale [64]. Satisfaction with care will be measured using a combination of four items [2, 5, 7, 8] from the Client Satisfaction Questionnaire (CSQ) [65], the Session Rating Scale (SRS) [66], and nine additional items tailored for Primary Care Behavioral Health (PCBH) settings to gauge patients’ perceptions and attitudes towards their care providers (see Supplementary materials C). The 5-item Treatment Credibility Scale evaluates how credible and effective participants perceive their treatment to be, enabling analyses of any link between perceived effectiveness and actual outcomes [67]. To evaluate treatment side effects, patients will complete an Adverse Events questionnaire, offered in both a concise (3 items) version at the FU4 and FU8 timepoints, and a more comprehensive version (9 items) at FU12, both available in Supplementary materials C. These allow patients to report and describe any unwanted treatment effects or events they may have experienced. Any adverse events requiring immediate action will be promptly reported to the responsible PCC.

#### Treatment content

Data on which interventions therapists use in each treatment modality will be extracted from medical records. Patients’ recollections of these interventions and which strategies they continue to use post-treatment will also be gathered qualitatively during the FU12 interview. Comparisons between treatment content in medical records, patient recollections and protocols (in GSH), as well as the broader evidence base (in BI), will be utilized to analyze variations in treatment outcomes. Medical records and interview responses will be coded using an inductive approach, where codes are developed directly from the data rather than from predefined categories. Double coding will be used to test inter-rater reliability, and any discrepancies will be resolved through discussion.

#### Feasibility, acceptability, and fidelity

##### Clinician experiences and preferences

Clinicians will be interviewed by a neutral research assistant to explore their perspectives on the extended assessment process, GSH, and BI. The interviews will examine their experiences, preferences, perceived differences between treatment approaches, and factors influencing treatment selection. Interviews will be transcribed and analyzed using thematic coding to identify key concepts, while each response is also rated on a numerical scale based on specific attributes (e.g., the usefulness, effectiveness, and adherence to each treatment model). Themes are developed inductively from the data, and coding is double-checked for inter-rater reliability. The interview guide is provided in the Supplementary Materials D.

##### Proportion of patients deemed suitable for GSH

The proportion of patients in the PCBH + GSH arm that are deemed suitable for GSH will be analyzed by both site and clinician. Potential sources of variation will be explored by examining patient characteristics, themes in clinician-reported reasons for not offering GSH to individual patients, and insights from clinician interviews. Retrospective assessments of suitability for structured interventions will also be conducted for patients who underwent extended assessments in practice, allowing for comparisons between the algorithm’s outcomes and real-life assessments.

##### Fidelity to GSH protocols and BI principles

Fidelity to each intervention will be assessed using data from medical records, the CRF, the FU12 interview, and free-form text responses from patient satisfaction and adverse event scales. To evaluate clinicians’ adherence to key treatment components, 5-point Likert scales will be used to interpret these data sources. For GSH, fidelity will be assessed based on factors such as the use of self-help materials, emphasis on core treatment components, alignment with the prescribed number of sessions, and consistency in treatment structure and focus. For BI treatments, key factors will include targeting changeable elements, maintaining focus, employing skill-building techniques, aligning with the evidence base, and ensuring clarity in treatment plans. These scales are provided in Supplementary Materials E, but may be somewhat adjusted prior to coding each treatment if the quality or content of the raw data differs from expectations. Fidelity will be analyzed both at the individual treatment level and averaged across clinicians and treatment models to assess overall adherence. To examine factors influencing variations in fidelity across sites, clinician interview responses will be analyzed as well as workload data. The Third Next Available Appointment (TNAA) metric, which measures the waiting time for a hypothetical patient seeking an appointment at the center, will serve as a proxy for clinician workload and will be used to assess whether higher workload correlates with lower fidelity to GSH protocols, or a reduced proportion of patients being deemed suitable for GSH.

#### Healthcare utilization and costs

The number of visits for each treatment type will be extracted from medical records. Treatment duration in hours will be calculated based on each site’s standard session length for in-person visits and phone or video consultations. Healthcare resource utilization and societal economic impact will be assessed using multiple measures. Direct treatment costs will be calculated based on the cost for self-help books, the number and duration of patient contacts, and the type of healthcare professional delivering the treatment. The Trimbos and iMTA questionnaire on Costs associated with Psychiatric Illness (TiC-P) will capture data on patient work productivity, including absenteeism due to sick leave, reduced efficiency while working, and productivity loss at home, as well as medication usage. The EQ-5D will be used to evaluate overall health-related quality of life for economic analyses.

### Automated algorithms

#### Automated assessment algorithm as a decision support tool

An automated algorithm will be used to assist clinicians in assessing the main problem area and determining suitability for GSH. In addition to the screening questionnaires outlined above, the algorithm utilizes vignettes featuring concise descriptions of each problem area targeted by GSH in the study (e.g., depression, worry/generalized anxiety disorder, social phobia, illness anxiety disorder, insomnia, stress and exhaustion disorder, panic disorder, and OCD), as well as two areas used to potentially disqualify patients from GSH treatment (life problems and distress due to medical conditions). These vignettes are available in Supplementary materials F. Patients are prompted to rate how accurately the description of each problem area reflects their problems (question A) on a scale from 0 to 4, where 0 represents “Not at all” and 4 represents “Completely”. They also rate the importance of each area and their motivation to address it (question B) on a scale from 1 to 4, where 1 represents “Not important at all” and 4 represents “Very important”. The rating for each vignette’s question A is multiplied by question B, generating a score between 0 and 16 for each problem area. After rating each problem area, patients select the one they consider most important to address, followed by their second priority. The algorithm then assigns 15 points to the top-rated problem area and 10 points to the second-most important one. Additionally, scoring above the cut-off on a given questionnaire (see Table 2), which indicates that a problem area may be significant and important to work with, adds 10 points for the respective problem area. Scoring below the cut-off yields 0 points. In this context, the cut-off does not indicate the likelihood of a diagnosis but rather whether the problem area is deemed to be relevant to the patient.

The automated algorithm calculates a total score between 0 and 51. A score of 51 can only be achieved if the same vignette is selected as both the most and second most important, which is permitted since it is possible for the patient to have one single, predominant issue. There is no formal cut-off score; however, a higher score indicates a greater likelihood that the patient is experiencing and willing to address a particular problem. Therefore, the results will not automatically determine the most suitable focus for the patient but serve as a decision-support tool for the clinician. High scores on the domains of life problems and distress due to medical conditions will be seen as indicators suggesting that GSH may not be the most suitable option, particularly if none of the GSH problem areas reach comparable levels. The algorithm does not incorporate the AUDIT-C and pain scores; instead, these scores are intended to be interpreted separately, alerting clinicians to potential issues that could diminish the suitability of GSH as a treatment option, even if one or more problem areas receive high points in the algorithm. Versions of this algorithm have previously been used in studies [68, 69].

#### Retrospective algorithm for assessing indication for structured treatment

To address research question (2) – which examines whether patients who would have been suitable for GSH but did not receive it face a risk of undertreatment – we have developed an assessment model to retrospectively evaluate patients’ indications for structured treatment related to mood disorders, anxiety, stress-related conditions, or insomnia among those randomized to the core PCBH model. The assessment model is designed to mirror the clinicians’ extended, diagnostic assessment as closely as possible, with the crucial distinction that categorization is based solely on self- and clinician-reported data, and patient treatment preferences are unknown since they are never offered the possibility to receive GSH. Patients in the core PCBH arm who are retrospectively assessed as not indicated for structured treatment are assigned to the CORE-N-BI group, while those who meet the criteria are placed in the CORE-I-BI group. The same retrospective assessment will be conducted for patients in the extended PCBH arm to allow for comparisons between the retrospective and real-life assessments; however, this will not impact their group assignment.

The retrospective assessment consists of four steps:

1. **Exclusion of patients clearly not indicated for structured treatment for the specified conditions**: These patients are identified based on their stated reasons for seeking help, reported health issues, and clinicians’ notes in the CRF.
2. **Evaluation of potentially suitable patients**: Patients identified as potentially suitable for structured treatment undergo evaluation using specific scoring criteria, similar to those outlined in the decision support tool described above. In brief, this evaluation includes results from the automated algorithm, AUDIT-C scores, and the 1-item pain scale. Points are assigned when patients exhibit high scores in relevant problem areas, while deductions are made for high scores in non-relevant domains or indications of substance abuse or pain that may interfere with treatment.
3. **Further evaluation of uncertain cases**: Patients with uncertain suitability are discussed with a supervisor and undergo additional evaluation, considering factors such as education level, health habits, and language difficulties.
4. **Final assessment for suitable patients**: Patients deemed suitable for structured treatment have their primary problem areas assessed. The most pertinent problem is identified based on the problem area with the highest score from the automated algorithm, in conjunction with the patient’s stated reason for seeking help.

A flow chart detailing the process of the retrospective assessment can be found in Supplementary materials F.

### Data management, monitoring and confidentiality

The study technical platform database BASS4, provided by Karolinska Core Facilities, will be used to gather and securely store data in encrypted form, behind two-factor authentication and accessible only to authorized research personnel. Self-report forms will primarily be completed online, meaning that the data is immediately stored in BASS4. Information collected through methods other than self-report forms (such as self-report forms for patients who wish to fill them in on paper) will be manually entered into BASS4 by research assistants, using predefined templates to ensure data integrity and adherence to expected value ranges. Several strategies will be used to enhance participant retention, including automated text reminders at each follow-up, phone calls from research assistants for incomplete forms, and the option to complete a shorter version including only key questionnaires or respond to the primary outcome measure (WHODAS-8) by phone.

Standardized coding practices will be employed for any necessary data encoding. Following data collection, all information will be pseudonymized and assigned unique identifiers, with access restricted to the principal investigator and university archivists. Other members of the project team will have access to pseudonymized data. Given the trial’s low anticipated risk to participants and the absence of mandatory data monitoring for psychological intervention trials in Sweden, the decision was made to forego a data monitoring committee due to financial reasons.

### Power analysis

The power analysis is based on the primary outcome and endpoint, specifically the sum of eight items from WHODAS-12 at FU12. In the pilot trial (manuscript in preparation), a preliminary effect of 0.18 (Cohen’s *d*) was observed between the two care models after eight weeks of treatment. However, at that time, some of the GSH interventions were still ongoing. Many self-help books in the study are structured into 10–12 chapters, completed at a pace of one per week, and many of the studies on the internet-based interventions have a duration of 10–12 weeks [32, 34, 35, 37, 44]. Given this, the endpoint of this full-scale RCT was adjusted to twelve weeks, under the assumption that patients would have finished their treatments, which may produce a somewhat larger effect. A previous trial using similar GSH interventions reported slightly stronger effects [24]. Considering these factors, we estimate an expected between-group effect size of *d* = 0.20. To achieve 80% power with an alpha of 0.05 while accounting for 20% missing data at FU12, a total sample size of 983 patients is required [70]. Recruitment will continue until the power target is reached, with efforts to identify and include new PCCs as necessary. The pilot study (manuscript in preparation) demonstrated an acceptable inclusion rate.

If the primary analysis fails to establish superiority, a secondary analysis will assess whether the core PCBH model is non-inferior to the extended model (PCBH + GSH). No established non-inferiority margin exists for WHODAS-12 in mental health patients in primary care, and even less so for the 8-item version used in this study. Therefore, although not ideal, we reference a previously established non-clinically relevant difference of *d* = 0.24 from a meta-analysis of patients receiving psychological treatment for depression [71]. However, as this threshold is based on disorder-specific measures and treatments, we anticipate that the transdiagnostic WHODAS will be less sensitive to change and will therefore set the non-inferiority margin to *d* = 0.20. To achieve 80% power with an alpha of 0.05, assuming 20% missing data, 775 patients will be required [70]. This falls within the target sample size for the primary analysis. The smaller sample size required for the non-inferiority analysis, despite the identical effect size used, is due to the use of a one-sided test.

For the secondary analyses of the subgroups of patients deemed suitable for structured treatment (CORE-I-BI vs. EXT-I-GSH), the between-group effect is expected to be larger, but the number of patients will also be lower. Given these uncertainties, a separate power analyses for this secondary research question is not performed.

### Data analysis plan

Due to the pragmatic nature of the study, where randomization occurs early – after patients have been identified and invited to participate, but before they are formally included in the trial – there is an increased risk of randomized participants during assessment or treatment being deemed unsuitable for both GSH and BI due to a need for specialized or primarily medical treatment. These participants will be excluded from most analyses but are expected to be randomly distributed between study arms. Another consequence of the pragmatic design is that patients may complete the pre-treatment measures up to a week after their initial visit to manage the usually very brief waiting time in PCBH. This could particularly impact within-group BI effects, as treatment begins during the first visit. To address this, a sensitivity analysis excluding late responses will be conducted, along with an analysis of missing data to evaluate its potential impact.

Several comparisons will be made to address the research questions. For research questions (1) and (3), we will conduct between-group comparisons on primary and secondary measures at the care model level, specifically comparing all participants in PCBH group with those in the PCBH + GSH group. To address research question (2), we will compare the subgroup of patients who received GSH (the EXT-I-GSH group) within the PCBH + GSH model with those who were retrospectively assessed as indicated for structured treatment but were randomized to the core PCBH arm and therefore did not receive GSH (the CORE-I-BI group). For the subgroup analysis examining whether GSH has a greater impact on conditions where the protocol is expected to have more specific effects (insomnia, panic disorder, social phobia, health anxiety disorder, and OCD) compared to conditions the effects are expected to be more general and non-specific (depression, GAD, and stress), analyses will evaluate whether patients in the former group show larger differences between GSH and BI than patients in the latter group. Research question (4) will be analyzed at both the care model and treatment type levels, as factors such as patient satisfaction, treatment content, patient behavior, and adverse events may be influenced by both the assessment and treatment strategies.

For research questions (5) and (6), the analysis groups will be defined based on treatment rather than the model of care. It is therefore essential to clarify how comparison groups are categorized, as they do not align fully with the randomized groups. There is only one subgroup of patients that receive GSH – the patients in the PCBH + GSH arm assessed by the clinician as suitable for GSH (the EXT-I-GSH group). In contrast, patients receiving BI can be divided in several groups, all presented in Table 3. Analyses will be conducted on various combinations of patients receiving BI to account for potential differences among these groups. For example, CORE-N-BI and EXT-N-BI may present with more complex symptoms or social challenges compared to CORE-I-BI – or alternatively, they may have less complex, transient life stressors. Additionally, the diagnostic assessment in the PCBH + GSH model may influence the effectiveness of BI in either direction, necessitating comparisons between patients who received BI in the core model and those in the extended model.

**Table 3:** Treatment-level analysis groups

	Core PCBH arm		PCBH + GSH arm	
	CORE-I-BI	CORE-N-BI	EXT-I-GSH	EXT–N-BI
**All patients receiving GSH**			X	
**All patients receiving BI**	X	X		X
**Receiving BI despite an indication for structured treatment**	X			
**Receiving BI in accordance with the indicated treatment**		X		X
**Receiving BI following an extended assessment confirming BI as appropriate**				X
**Receiving BI after the contextual interview**	X	X		

Notes. CORE-I-BI = Patients receiving brief interventions (BI) within the core PCBH model who were retrospectively assessed as suitable for structured treatment

CORE-N-BI = Patients receiving brief interventions (BI) within the core PCBH model who were retrospectively assessed as not suitable for structured treatment

EXT-I-GSH = Patients receiving guided self-help (GSH) after undergoing an extended diagnostic assessment within the PCBH + GSH model, where they were deemed suitable for structured treatment

EXT-N-BI = Patients receiving brief interventions (BI) after an extended diagnostic assessment within the PCBH + GSH model, where they were not deemed suitable for structured treatment

In question (5), the difference in variation of treatment outcomes due to intervention (primarily comparing EXT-I-GSH with CORE-I-BI) will be evaluated by testing if the variation in FU12-scores differs between the two groups using an F-test. To put the difference in variation after treatment in context, the same type of F-test will be made on PRE-scores. The first question in (6) focuses solely on BI and how the therapists’ choice of methods affects outcome. This question requires rating all identified therapeutic methods based on the extent to which they are deemed to have an empirically supported effect for the condition they address. The protocol for these ratings will be developed once all therapeutic methods are identified and prior to exploring their relation to outcomes. The main analysis will be made on all patients receiving BI, regardless of subgroup. The second question in (6) examines potential differences between the subgroups receiving BI. For the third question in (6), only patients receiving GSH will be included, and ratings of therapist adherence will be used to explore its overall relationship to outcomes, as well as for subgroups of patients where GSH manuals with expected specific versus non-specific effects are used. For the final question in (6), relationships between treatment content and outcomes will be explored for all patients, regardless of care model or subgroup.

For research questions (7), (8), and (9), the focus shifts to exploring the feasibility and fidelity of implementing the PCBH + GSH model at scale across multiple PCBH PCCs. To address research question (7), we will examine the proportion of patients who are deemed suitable for GSH following the extended diagnostic assessment. A hierarchical model using a binomial distribution will be used to evaluate whether the suitability rate varies significantly between as well as within centers over time, and to explore any factors contributing to these variations. In research question (8), we will use Hierarchical Linear Models to investigate how patient-related outcomes are affected by interventions and vary across centers and therapists, as well as the factors contributing to these differences. This will help identify potential barriers and facilitators for the successful implementation of both the core and extended PCBH models across different settings. Finally, research question (9) will explore the fidelity of both GSH and BI treatments. Specifically, we will examine how closely clinicians adhere to GSH principles within the PCBH framework, particularly in areas where GSH and PCBH principles may conflict, as well as generally assess the quality of the BI interventions. Fidelity scores will be analyzed descriptively to determine the frequency of low, good-enough and good fidelity ratings. A *t*-test will be used to evaluate whether the mean fidelity score is 3 (good-enough) or higher. Additionally, fidelity will be analyzed across clinicians and PCCs using chi-square tests and logistic regression to identify variability and the factors contributing to it. This will help assess the overall quality of treatment delivery and identify areas that may need further support or adjustment for large-scale implementation.

The primary statistical methods employed will be Hierarchical Linear Models and Generalized Estimating Equations. Missing data will be handled using multiple imputation techniques that align with the missing data mechanism, while accounting for the multilevel structure of the data. Imputation models will be specified to reflect the underlying data structure, provided the complexity does not hinder model convergence. The analyses will consider the hierarchical structure of the data, such as nesting patients under therapists under PCCs for patient outcomes. The variance of each grouping level in the models will be calculated as the intra-class correlation (ICC) metric. An ICC > 0.10 will be used as a cutoff for the relevance of including it in the final model.

The non-inferiority analysis will be conducted by estimating the mean differences and standard errors at FU12, using Rubin’s rule after performing multiple imputations for missing data. A one-sided 95% CI (corresponding to a 90% two-sided CI) will be calculated for the estimated mean difference. To determine the outcome, we will compare the lower bound of the confidence interval with the non-inferiority margin of *d* = 0.20. If the lower bound of the CI is above this margin, non-inferiority will be established, in line with prior recommendations [72]. For research question (4), which hypothesizes that GSH protocols with assumed specific effects may demonstrate superior outcomes compared to BI, we will include a dummy variable in the model to represent the type of GSH protocol used (specific or non-specific).

For Health Economic Analyses in question (3), effectiveness will be assessed by constructing quality-adjusted life-years (QALYs) based on EQ-5D. Incremental cost-effectiveness ratios (ICERs) will be calculated, and Monte Carlo simulation with non-parametric bootstrapping will be conducted to address the uncertainty of the ICER point estimates. Cost-effectiveness acceptability curves (CEACs) will be employed to examine cost-effectiveness across varying willingness-to-pay scenarios.

## Discussion

In this study protocol, we present a comprehensive framework for evaluating the effectiveness of the PCBH + GSH model compared to the core PCHB model. Additionally, we aim to increase knowledge of which patient groups benefit most from which type of intervention as well as test an assessment model that could be integrated into standard healthcare practice. Our aim is to generally expand the evidence base for PCBH and specifically explore whether GSH can complement the treatment options. We also seek to investigate the cost-effectiveness associated with the PCBH + GSH model compared to core PCBH and evaluate several aspects related to care processes and implementation. These findings will offer valuable insights for optimizing healthcare resource allocation and mental health service delivery in primary care settings.

However, certain difficulties should be acknowledged. Conducting clinical studies within routine healthcare settings presents inherent challenges, and implementing GSH – a structured treatment grounded in the medical model – within organizations accustomed to the contextually based, pragmatic PCBH model may introduce additional complexities. This may be more pertinent during periods of time and resource constraints in the PCC. While PCBH operates under a population-based healthcare approach, marked by flexible problem-solving in the face of constraints, ESTs adheres to principles aimed at following protocols maximize treatment effects for individual patients [29]. In a PCC where both treatment options are being investigated, divergent opinions may emerge regarding the appropriate course of action when resources are insufficient: either adjusting the duration or intensity of treatment to maintain high availability or reducing availability and providing a complete GSH intervention within the timeframe. In this study, where clinicians ultimately decide whether a diagnostic assessment ends with a patient being offered GSH or not, it may also impact how many patients are offered GSH. This discrepancy could potentially impact fidelity to either the GSH protocols or the PCBH principles. These issues are considered research questions in their own right, as they could offer valuable insights into the broader feasibility of the PCBH + GSH model, in addition to posing potential threats to the internal validity of the clinical trial. Other limitations of this study, as previously mentioned, include the early randomization procedure, which occurs prior to formal study inclusion, and the potential delay in patients completing the pre-treatment measures, which may be submitted up to a week after their initial visit. Additionally, certain effects, such as whether the implementation of GSH reduces availability, cannot be fully evaluated, as each PCC offers both the core and extended models simultaneously. This limits the generalizability of the findings regarding what would occur if only one model were implemented at a given PCC.

In conclusion, the study described in this protocol represents a significant advancement in integrated mental health care delivery in primary care. Through rigorous evaluation of PCBH and a possible integration of GSH in the PCBH model, we aim to inform evidence-based practice and policy development in primary care mental health.

### Current trial status

Recruitment began on June 14th, 2021, and is ongoing, following the outlined power analysis. The anticipated end date for recruitment is May 2025.

## Supplementary Information

Below is the link to the electronic supplementary material.

Supplementary Material 1

Supplementary Material 2

Supplementary Material 3

Supplementary Material 4

Supplementary Material 5

Supplementary Material 6

Supplementary Material 7

Supplementary Material 8

Supplementary Material 9

## Data Availability

No datasets were generated or analysed during the current study.

## Abbreviations

BI: Brief interventions
CI-I-BI: Core PCBH patients indicated for structured treatment but receiving BI
CORE-N-BI: Core PCBH patients not indicated for structured treatment receiving BI
CBT: Cognitive Behavioral Therapy
EST: Empirically Supported Psychological Treatments
EXT-I-GSH: Extended PCBH patients indicated for structured treatment receiving GSH
EXT-N-BI: Extended PCBH patients not indicated for structured treatment, receiving BI
GSH: Guided self-help
PCBH Core: Primary Care Behavioral Health model
PCBH + GSH: Extended Primary Care Behavioral Health model
PCC: Primary Care Center
