# Supplementary material for: Evaluation of Primary Care Behavioral Health (PCBH) with guided self-help CBT as a treatment option – a protocol of a single-blind randomized multicenter trial (KAIROS)
Source: BMC Health Serv Res. 2025 Sep 23;25:1208. doi: 10.1186/s12913-025-13232-4 (PMC12455819; doi:10.1186/s12913-025-13232-4)
Supplement: Supplementary file 1 — Supplementary Material 1 [file 12913_2025_13232_MOESM1_ESM.docx]

Supplementary materials A

Sociodemographic and clinical data forms

# Background questions 10-items

**1. What is your current relationship/marital status?**

- Married, cohabiting, or in a registered partnership
- In a committed relationship (living apart)
- Divorced or separated
- Widow or widower
- Single
- Other, namely...

**2. What is your highest level of completed education?**

- Primary school, elementary school, middle school, or similar
- 2-year high school or vocational school
- 3- or 4-year high school
- Folk high school^[[1]](#footnote-1)^ or similar
- University or college, less than 3 years
- University or college, 3-4 years
- University or college, 5 years or more

**3. What is your current occupation?**
(Multiple options can be selected. If you are currently on sick leave but have been away from work for less than 3 months, only indicate the occupation you have when not on sick leave.)

- Employed
- Self-employed
- On leave or parental leave
- Studying, interning
- In a labor market program
- Unemployed
- Retired
- On disability or activity allowance (early retirement or disability pension)
- On long-term sick leave (more than 3 months)
- Managing your household
- Other, namely...

**4. What is your profession?**
If you are not currently working, state the profession or job you have primarily had. If you have multiple jobs, specify the main one. Provide as detailed a description as possible. For example, instead of "teacher," write "elementary school teacher"; instead of "nurse," write "district nurse"; instead of "consultant," specify the field in which you are consulting.

**5. To what extent are you currently engaged in work, studies, or self-employment?**

- 100%
- 75–99%
- 50–74%
- 25–49%
- 1–24%
- 0%

**6. Would you or your household be able to pay an unexpected expense of 13,000 SEK within a month without borrowing or asking for help?**

- No
- Yes

**7. In the past 12 months, have you experienced difficulties covering regular expenses such as food, rent, bills, etc.?**

- No
- Yes, once
- Yes, multiple times

**8. Do you have someone with whom you can share your innermost feelings and confide in?**

- No
- Yes

**9. Can you get help from someone if you have practical problems or are sick? For example, getting advice, borrowing items, help with groceries, repairs, etc.?**

- No
- Yes

**10. Who do you live with (at least half the time)?**
(Multiple options can be selected.)

- No one
- Parents and/or siblings
- Spouse, cohabiting partner, or partner
- Other adults
- Children

# Questions about health issues and care 6-items

**1.** What is the main reason for your visit to the primary care center? Please describe briefly in your own words.

**2.** How long have you been experiencing the problems described above?

- Less than 1 month
- 1–3 months
- 3–6 months
- 6–12 months
- 1–2 years
- 2–10 years
- More than 10 years

**3.** Do you have any other health issues or problems for which you are currently seeking help? Do not include health issues that you have but are not seeking help for at the moment.

- No
- Yes
  If yes, please specify:

**4.** Are you currently undergoing any form of psychological treatment, counseling, therapy, group therapy, or support contact?

- No
- Yes
  If yes, please briefly describe each treatment and what it aims to address:

**5.** Have you previously undergone psychological treatment, counseling, therapy, group therapy, or had a support contact?

- No
- Yes, a total of 1–5 visits/sessions/meetings
- Yes, a total of 6–10 visits/sessions/meetings
- Yes, a total of 11–15 visits/sessions/meetings
- Yes, a total of 16–20 visits/sessions/meetings
- Yes, more than 21 visits/sessions/meetings

**6.** If yes, was it for the same issues you are seeking help for now?

- No
- Yes
- Have not had treatment before

1. A type of educational institution in Sweden, primarily catered to adult learners, that offers a wide range of courses, often focused on personal development, vocational training, or preparatory studies for higher education. They do generally not grant academic degrees. [↑](#footnote-ref-1)
