# Supplementary material for: Evaluation of Primary Care Behavioral Health (PCBH) with guided self-help CBT as a treatment option – a protocol of a single-blind randomized multicenter trial (KAIROS)
Source: BMC Health Serv Res. 2025 Sep 23;25:1208. doi: 10.1186/s12913-025-13232-4 (PMC12455819; doi:10.1186/s12913-025-13232-4)
Supplement: Supplementary file 2 — Supplementary Material 2 [file 12913_2025_13232_MOESM2_ESM.docx]

Supplementary materials B

FU12 interview guide

# The interview

1: Introduction
“Hello! I’m looking for *[participant’s name]*. My name is *[your name]* and I’m calling from the KAIROS research project. I’m calling to follow up on how things have been going for you, three months after your first contact with *[patient’s primary care center]*.”

“Can we go ahead now?” *or*
“We have scheduled an interview now; can we start?” *or*
“Is there another time that works better for you?”

“OK, great! I will be reading some things verbatim so that all interviewees get the same questions. Shall we start?”

## 2: WHODAS-8 (if not filled in online)

“I noticed we haven’t received all your responses to the online forms. I’ll start by asking the 8 most important questions from there. These questions are about difficulties related to your health condition. By health condition, we mean diseases and other health problems that may be short-term or long-term, injuries, mental or emotional issues, and problems with alcohol or drugs. Think back over the past 30 days and answer these questions based on how difficult it has been for you, on average, to perform the following activities because of your health condition.

**In the past 30 days, how much difficulty did you have in doing household chores due to your health condition?**
None, Mild, Moderate, Severe, Extreme, or cannot do?

**In the past 30 days, how much difficulty did you have in learning a new task, for example, learning how to get to a new place?**
None, Mild, Moderate, Severe, Extreme, or cannot do?

**In the past 30 days, how much difficulty did you have in joining community activities (for example, festivities, religious or other activities) in the same way as anyone else can?**
None, Mild, Moderate, Severe, Extreme, or cannot do?

**In the past 30 days, how much difficulty did you have in managing the emotional effects of your health problems?**
None, Mild, Moderate, Severe, Extreme, or cannot do?

**In the past 30 days, how much difficulty did you have in concentrating on doing something for ten minutes?**
None, Mild, Moderate, Severe, Extreme, or cannot do?

**In the past 30 days, how much difficulty did you have in dealing with people you do not know?**
None, Mild, Moderate, Severe, Extreme, or cannot do?

**In the past 30 days, how much difficulty did you have in maintaining a friendship?**
None, Mild, Moderate, Severe, Extreme, or cannot do?

**In the past 30 days, how much difficulty did you have in doing your day-to-day work or studies?**
None, Mild, Moderate, Severe, Extreme, or cannot do?

“Thank you! It would be great if you could answer the questions online when we finish this interview. There are important questions there that I don’t have time to ask on the phone. Do you know where to log in to answer the questions? Would you like me to send or go over how to log in again? Could you do that after our call?”

*[If the patient needs help logging in or needs paper forms, refer to SOP.]*

3: Treatment goals
“I’m going to ask you about what you remember from and what you’ve done in your treatment.

1a. “Did you and your therapist set goals and plans for the treatment?”
❒ I definitely remember no goals/plans were made
❒ I don’t think goals/plans were made
❒ I think one or more goals/plans were made
❒ I definitely remember one or more goals/plans were made

1b. *[If the patient answers affirmatively.]*

“Tell me about which goals and plans you set.”

*[Write down in Table 1.]*

1c. *[If the patient gives a vague answer or misses a plan listed in the medical record (only if you have access to the patient’s record).]*

 “In your medical record, this goal is listed *[read aloud].* How well do you remember it on a scale from 0–4, where 0 means ‘I don’t remember it at all’ and 4 means ‘I remember it exactly’?”

*[Write down in Table 1, including your assessment of how well the patient’s and the medical record’s descriptions align (0–4).]*

4: Changes
2a. “What changes have you made in your daily life or life in general since your first visit to the primary care center three months ago? For example, have you changed habits or done things differently than before?”

*[Write down in Table 2, including your rating of how related the change is to the treatment and the goals you previously discussed (0-3).]*

2b. “How much effort or time have you put into making the change, where 0 means none and 4 means a lot of effort or time?”

*[Write down in Table 2.]*

2c. “Now I wonder if this change has made you feel better or worse. Of the following alternatives, which do you think fits best?”
❒ 1 – Very large deterioration
❒ 2 – Large deterioration
❒ 3 – Slightly worse
❒ 4 – No change
❒ 5 – Slightly improved
❒ 6 – Large improvement
❒ 7 – Very large improvement

*[Write down in Table 2.]*

*[Repeat for each stated change.]*

5: Negative Events

3a. **“**During the course of your treatment, have you experienced any unwanted event that you believe was caused by the treatment? Or have you had any unwanted effects from the treatment?”

*[If the participant does not understand the question, provide examples.]*

“For example, feeling worse in some way, the treatment not working as expected, a poor relationship with the therapist, or experiencing negative effects in daily life.”

̆❒ No, no negative event
̆❒ Yes

3b. *[If yes: ask the patient to describe each event. For each event, ask the following.]*

- What was the event?
- How often/how long did it last?
- How did this affect you at the time?
- How does this affect you today?

3c. *[Based on the patient’s freely formulated response, rate the severity of the event using the following scale. If you are uncertain, choose the higher alternative and allow the patient to adjust as needed.]*

̆ 0 – Mild
̆ 1 – Moderate, distressing
̆ 2 – Severe, requiring intervention
̆ 3 – Very severe, with lasting consequences
̆ 4 – Extremely severe, life-threatening

*[Then verify your rating with the patient.]*

- (0) “This was something that affected you at the time but hasn’t had much impact on you, is that correct?”
- (1) “This was a rather distressing event that you have thought about afterward, is that correct?”
- (2) “This was a serious event that required some kind of action, is that correct?”
- (3) “This was a very serious event that has had lasting consequences for you, is that correct?”
- (4) “This was an extremely serious event that posed a threat to your life, is that correct?”

*[Adjust your rating up or down as needed based on the patient’s response.]*

3d. *[Based on the patient’s freely formulated response, rate the event’s connection to the treatment using the following scale. If you are uncertain, choose the higher alternative and allow the patient to adjust as needed.]*

̆ 0 – Not at all related to the treatment, unrelated
̆ 1 – Probably unrelated
̆ 2 – Possibly related
̆ 3 – Probably related
̆ 4 – Related

*[Then verify your rating with the patient.]*

- (0) ... it was not related to your treatment at the healthcare center, is that correct?
- (1) ... it was probably not related to your treatment at the healthcare center, is that correct?
- (2) ... it was possibly related to your treatment at the healthcare center, is that correct?
- (3) ... it was probably related to your treatment at the healthcare center, is that correct?
- (4) ... it was definitely related to your treatment at the healthcare center, is that correct?

*[Adjust your rating up or down as needed based on the patient’s response.]*
