# Supplementary material for: Evaluation of Primary Care Behavioral Health (PCBH) with guided self-help CBT as a treatment option – a protocol of a single-blind randomized multicenter trial (KAIROS)
Source: BMC Health Serv Res. 2025 Sep 23;25:1208. doi: 10.1186/s12913-025-13232-4 (PMC12455819; doi:10.1186/s12913-025-13232-4)
Supplement: Supplementary file 3 — Supplementary Material 3 [file 12913_2025_13232_MOESM3_ESM.docx]

Supplementary materials C

Unpublished patient-rated report forms

# Adverse events 3-item

The following questions are asked to determine if the care, treatment, or wait for care or treatment you received has led to any unwanted events or effects. The purpose here is to find out if the treatment you received has caused any short- or long-term side effects, or if you have deteriorated or experienced other unwanted effects while waiting for treatment. You can describe as many unwanted events or effects as you wish in free text.

1. During the course of your treatment, have you experienced any unwanted events or effects that you believe are due to the treatment? Or, while waiting for treatment, have you experienced any unwanted effects or deterioration? [Yes, No]
2. Describe the unwanted events or effects. Also indicate when during the treatment or waiting period these events/effects occurred, how often they occurred, and how long each event/effect lasted. [Free text]
3. How negatively do you believe these unwanted events or effects impacted your well-being when they occurred? [Did not affect me at all, Affected me slightly negatively, Affected me moderately negatively, Affected me very negatively]”

# Adverse events 9-item

The following questions are asked to find out if the care, treatment, or waiting for care or treatment you have received has led to any adverse events or effects. The aim here is to determine if the treatment you received can cause any short- or long-term side effects, or if you have worsened or experienced other adverse effects while waiting for treatment. You have the opportunity to provide detailed information on two adverse events or effects. If you have experienced more than two adverse events or effects, describe the two that have had the greatest negative impact on your well-being and use the lines at the bottom to describe other adverse events and adverse effects.

1. During the course of treatment, have you experienced any adverse events that you believe are due to the treatment or received any adverse effects from the treatment? Or have you experienced any adverse effects or deterioration while waiting for treatment? [Yes, No]
2. Describe the adverse event or adverse effect. Also specify when during the treatment/waiting period these events/effects occurred, how often they occurred, and how long each event/effect lasted. [Free text]
3. How negatively do you believe these adverse events or adverse effects affected your well-being when they occurred? [Did not affect me at all, Affected me slightly negatively, Affected me moderately negatively, Affected me very negatively]
4. How related do you perceive the adverse event to be to the treatment you received or the fact that you were waiting for treatment? [Unrelated, Probably unrelated, Possibly related, Probably related, Related]
5. During the course of treatment, have you experienced any additional adverse events that you believe are due to the treatment/waiting period or received any adverse effects from the treatment/waiting period? [Yes, No]
6. Describe the adverse event or adverse effect. Also specify when during the treatment/waiting period these events/effects occurred, how often they occurred, and how long each event/effect lasted. [Free text]
7. How negatively do you believe these adverse events or adverse effects affected your well-being when they occurred? [Did not affect me at all, Affected me slightly negatively, Affected me moderately negatively, Affected me very negatively]
8. How related do you perceive the adverse event to be to the treatment you received? [Unrelated, Probably unrelated, Possibly related, Probably related, Related]
9. If during the course of treatment or waiting period you have experienced any additional adverse events that you believe are due to the treatment or received any adverse effects from the treatment or waiting period, describe them here. Specify when these events/effects occurred, how often they occurred, how long each event/effect lasted, how negatively they affected your well-being then, and how negatively they affect your well-being now. [Free text]”

# Assessment of your mental health care at the PCC 9-items

Each item is rated 0-5 on the scale [Never true, Very rarely true, Sometimes true, Often true, Almost always true, Always true].

1. The staff at the health center take my problems seriously.
2. The staff at the health center care about me.
3. During each visit, the staff show interest in my life situation and how I am doing.
4. The staff offer suggestions on how I can be helped.
5. The health center has the resources available for the staff to help me.
6. The care I receive contributes to my condition slowly but surely improving.
7. I know how to help the staff help me.
8. The staff and I share the same understanding of my condition.
9. I can recommend the care I receive to other patients with similar issues.
10. Think about how your past week has been. How many days in the past week have you:

# DAily Routines for Well-being INventory (DARWIN) 11-item

Think about how your past week has been. How many days in the past week have you:

**1a.** Spent at least **10 consecutive minutes** doing **moderately strenuous daily activities** that increase your heart rate and breathing slightly?
*For example, walking, cycling, light yoga, or physically demanding household chores.*
**[ ] days out of 7**

**1b.** Spent at least **30 consecutive minutes** doing **moderately strenuous daily activities** that increase your heart rate and breathing slightly?
*For example, walking, cycling, light yoga, or physically demanding household chores.*
**[ ] days out of 7**

**2a.** Spent at least **10 consecutive minutes** doing **cardio or strength training** that significantly increases your heart rate and makes you short of breath?
*For example, running, aerobic exercise, ball sports, strength training, squats, or push-ups.*
**[ ] days out of 7**

**2b.** Spent at least **30 consecutive minutes** doing **cardio or strength training** that significantly increases your heart rate and makes you short of breath?
*For example, running, aerobic exercise, ball sports, strength training, squats, or push-ups.*
**[ ] days out of 7**

**3.** Eaten all the meals you usually want and need, without skipping or postponing meals?
*For most people, this means eating breakfast, lunch, and dinner.*
**[ ] days out of 7**

**4.** Eaten proper meals (not mainly snacks, candy, fruit, or similar) that keep you full and give you the energy to do what you need and want to do?
**[ ] days out of 7**

**5.** Slept at night and stayed awake during the day, meaning you went to bed before midnight and woke up before 9 AM?
**[ ] days out of 7**

**6.** Spent at least **30 minutes outdoors in daylight**?
**[ ] days out of 7**

**7.** Gone to bed and woken up at a **consistent time** (+/- 1 hour)?
*Do not differentiate between workdays and days off. If, for example, you stay up late or sleep in on weekends but go to bed and wake up at the same time on weekdays, mark* ***5****.*
**[ ] days out of 7**

**8.** **Consciously deprioritized sleep** or been unable to sleep as usual due to external circumstances?
**[ ] days out of 7**

**9.** Done something in your free time that helped you **relax, have fun, take your mind off stress and responsibilities, get a break, make progress on something, or develop a skill**?
For example, hobbies, spending time in nature, socializing, taking a course, or learning something new.
**[ ] days out of 7**

**10.** Had **meaningful or enjoyable social interactions** with other people?
**[ ] days out of 7**

**11.** How long have your habits been like this?
☐ Only the past week
☐ The past month
☐ The past two months
☐ 3–5 months
☐ 6–11 months
☐ One year
☐ More than one year

# **Obessive Compulsive Disorder 3-item**

**Obsessive Thoughts (Obsessions) Are:**

- Recurrent thoughts, impulses, or mental images that keep appearing despite attempts to ignore or get rid of them.
- Unwanted, repulsive, inappropriate, intrusive, or distressing.
- Cause anxiety and discomfort, leading to attempts to suppress them through thoughts or actions.

**Examples:**

- A fear of being dirty, contaminated, or having germs on you.
- A fear of infecting others.
- A fear of harming someone unintentionally.
- A fear of acting on an impulse in an inappropriate way.
- A fear of being responsible for something going wrong.
- Compulsive sexual thoughts, images, or impulses.
- The need to hoard or collect items.
- Religious obsessions.

### **Compulsive Actions (Compulsions) Are:**

- Actions performed to prevent something bad from happening or to reduce anxiety or discomfort.
- Actions that feel necessary to perform repeatedly due to a fixation or the need to follow a strict rule.
- Actions that are excessive or irrational.

**Examples:**

- Excessive washing and cleaning.
- Counting or checking things repeatedly.
- Repeating, collecting, or arranging items.
- Superstitious rituals.
- Silently repeating calming phrases, words, or counting to oneself.

### **Assessment Questions:**

1. **Have you experienced obsessive thoughts in the past week, and if so, how disturbing/distressing have they been?**
   - Not at all disturbing/distressing
   - Mildly disturbing/distressing
   - Moderately disturbing/distressing
   - Severely disturbing/distressing
   - Extremely disturbing/distressing
2. **Have you engaged in compulsive behaviors in the past week, and if so, how disturbing/distressing have they been?**
   - Not at all disturbing/distressing
   - Mildly disturbing/distressing
   - Moderately disturbing/distressing
   - Severely disturbing/distressing
   - Extremely disturbing/distressing
3. **Have you experienced obsessive thoughts and/or compulsive behaviors in the past week, and if so, how much time have they taken up?**
   - No obsessive thoughts or compulsive behaviors
   - Obsessive thoughts or compulsive behaviors rarely, occupying less than 1 hour/day
   - Obsessive thoughts or compulsive behaviors often, occupying 1–3 hours/day
   - Obsessive thoughts or compulsive behaviors very often, occupying more than 3 hours/day
   - Obsessive thoughts or compulsive behaviors almost constantly, occupying more than 8 hours/day
