# Supplementary material for: Evaluation of Primary Care Behavioral Health (PCBH) with guided self-help CBT as a treatment option – a protocol of a single-blind randomized multicenter trial (KAIROS)
Source: BMC Health Serv Res. 2025 Sep 23;25:1208. doi: 10.1186/s12913-025-13232-4 (PMC12455819; doi:10.1186/s12913-025-13232-4)
Supplement: Supplementary file 4 — Supplementary Material 4 [file 12913_2025_13232_MOESM4_ESM.docx]

Supplementary materials D

Interview guide for clinicians

1. What do you think about the extended assessment?

   a. If the therapist does not bring this up themselves: Do you feel that the extended assessment provides more information than the assessment you typically perform?

   b. If the therapist does not bring this up themselves: How did you feel about using the information you received from BASS, that is, the patient's ratings on scales and different problem descriptions?

   c. If the therapist does not bring this up themselves: Can the extended assessment be adjusted in any way?
2. What do you think about focused interventions as a treatment method?
3. What do you think about guided self-help as a treatment method?
4. Do you feel that there is a clear difference between your treatment interventions when you use focused interventions versus guided self-help? If so, what is the difference?

   a. If the therapist does not bring this up themselves: To what extent do you feel that the active interventions in guided self-help treatments are the same as in your focused interventions? For example, behavioral activation, exposure, mindfulness, etc.

   b. If the therapist does not bring this up themselves: Have you changed anything in the content of your focused interventions after receiving training in and reading self-help literature?
5. Do you feel that you have been able to offer guided self-help to suitable patients in the way you were instructed to do?

   a. If the therapist does not bring this up themselves: Have factors unrelated to the patient's issues or characteristics, such as time constraints and high demand, influenced your choice?
6. What factors do you feel most influence whether you choose focused interventions or guided self-help?
