# Supplementary material for: Evaluation of Primary Care Behavioral Health (PCBH) with guided self-help CBT as a treatment option – a protocol of a single-blind randomized multicenter trial (KAIROS)
Source: BMC Health Serv Res. 2025 Sep 23;25:1208. doi: 10.1186/s12913-025-13232-4 (PMC12455819; doi:10.1186/s12913-025-13232-4)
Supplement: Supplementary file 5 — Supplementary Material 5 [file 12913_2025_13232_MOESM5_ESM.docx]

Supplementary materials E

Interview guide for clinicians

# Brief Intervention (BI) Quality Assessment

1. **Was the treatment primarily focused on one specific problem area?**
   - 1: Strongly Disagree (Treatment was unfocused and addressed multiple unrelated issues)
   - 2: Disagree (Focus was inconsistent, with significant deviations from the intended problem area)
   - 3: Neutral (Some focus on a specific problem area, but with noticeable deviations)
   - 4: Agree (Mostly focused on one specific problem area, with minor deviations)
   - 5: Strongly Agree (Focused solely on one specific problem area)
2. **Did the clinician focus on problem area(s) that are changeable, and conceptualize the problem in a way that makes it possible to address and improve?**
   - 1: Strongly Disagree (No focus on changeable factors or conceptualizing the problem in a way that makes change possible)
   - 2: Disagree (Minimal focus on changeable factors or conceptualization, with significant focus on factors that cannot be changed)
   - 3: Neutral (Some focus on changeable factors, but not framed in a way that facilitates change)
   - 4: Agree (Mostly focused on changeable factors, though some aspects of the problem were not fully conceptualized in a way that allows for change)
   - 5: Strongly Agree (Focused entirely on changeable factors and framed the problem in a way that makes change possible)
3. **Did the intervention include skill-building techniques or encourage behavioral changes, such as using experiential methods (e.g., mindfulness, exposure)?**
   - 1: Strongly Disagree (No skill-building or behavioral focus)
   - 2: Disagree (Minimal skill-building or behavioral focus)
   - 3: Neutral (Some relevant skill-building, but not consistently emphasized or integrated)
   - 4: Agree (Skill-building and behavioral change elements were mostly incorporated)
   - 5: Strongly Agree (Skill-building and behavioral change were thoroughly incorporated)
4. **Did the clinician encourage patient empowerment by making sure the patient had a clear and shared understanding of the problem, with a plan they could work with independently (e.g., clear communication, actionable plans, and guidance on how to schedule follow-up appointments)?**
   - 1: Strongly Disagree (No clear or shared understanding of the problem, and no actionable plan for patient empowerment provided)
   - 2: Disagree (Limited clarity and shared understanding of the problem, with minimal actionable steps for patient empowerment)
   - 3: Neutral (Some clarity and shared understanding of the problem, but the actionable plan for patient empowerment could have been more thorough or clearly defined)
   - 4: Agree (Clear and shared understanding of the problem, with an actionable plan that supports patient empowerment, though some details may be lacking or need more emphasis)
   - 5: Strongly Agree (Clear and shared understanding of the problem resulting in an actionable plan for patient empowerment)
5. **Did the clinician use interventions that were clearly supported by evidence, based on current research or best practices?**
   - 1: Strongly Disagree (The intervention lacked any clear evidence or research support)
   - 2: Disagree (Minimal or unclear evidence supporting the intervention)
   - 3: Neutral (Some evidence or research was referenced, but its application was not fully clear)
   - 4: Agree (The intervention was mostly supported by strong evidence or best practices)
   - 5: Strongly Agree (The intervention was fully supported by well-established evidence or current best practices)
6. **Was there a clear differentiation between BI and other treatment types (e.g., GSH) in the delivery of treatment?**
   - 1: Strongly Disagree (No clear differentiation)
   - 2: Disagree (Some differentiation, but unclear)
   - 3: Neutral (Some effort made, but confusion remains)
   - 4: Agree (Clear differentiation, with minor overlaps)
   - 5: Strongly Agree (Completely clear and consistent differentiation)

# Guided self-help (GSH) Quality Assessment

1. **Were the key components of GSH (e.g., use of self-help materials, psychoeducation, follow-up on reading) incorporated into the treatment?**
   - 1: Strongly Disagree (Key components were mostly absent)
   - 2: Disagree (Key components were inconsistently incorporated)
   - 3: Neutral (Most key components were present, but with notable gaps)
   - 4: Agree (Key components were mostly incorporated)
   - 5: Strongly Agree (Key components were fully and consistently incorporated)
2. **Were the core treatment components of GSH, as outlined in the manual (e.g., exposure for anxiety), adequately emphasized during the guiding sessions?**
   - 1: Strongly Disagree (Core components were not included)
   - 2: Disagree (Core components were inadequately emphasized)
   - 3: Neutral (Core components were included but with notable gaps or inconsistencies)
   - 4: Agree (Core components were mostly emphasized)
   - 5: Strongly Agree (Core components were consistently emphasized)
3. **Was the number of sessions delivered in line with the GSH protocol?**
   - 1: Strongly Disagree (Sessions were far fewer or significantly exceeded protocol)
   - 2: Disagree (Some deviations, with a substantial mismatch in session count)
   - 3: Neutral (Sessions were within an acceptable range but with some inconsistencies)
   - 4: Agree (Sessions were mostly aligned with protocol)
   - 5: Strongly Agree (Sessions exactly followed the protocol)
4. **Was the spacing of sessions consistent with the GSH protocol?**
   - 1: Strongly Disagree (Significant deviation from recommended spacing)
   - 2: Disagree (Moderate deviation from spacing)
   - 3: Neutral (Some inconsistencies, but mostly followed recommended spacing)
   - 4: Agree (Spacing was mostly consistent with protocol)
   - 5: Strongly Agree (Fully adhered to recommended session spacing)
5. **Were there significant deviations from the GSH protocol (e.g., major changes to session structure, omission of key components, or substantial shifts in treatment focus)?**
   - 1: Strongly Disagree (Major deviations from protocol, substantial modifications)
   - 2: Disagree (Some major deviations, affecting core structure or focus)
   - 3: Neutral (Minor inconsistencies in structure or focus)
   - 4: Agree (Mostly followed the protocol with only minor, infrequent deviations)
   - 5: Strongly Agree (No significant deviations; strictly adhered to protocol)
6. **Was there clear differentiation between GSH and BI in treatment delivery?**
   - 1: Strongly Disagree (No clear differentiation between GSH and BI)
   - 2: Disagree (Some differentiation, but not clear)
   - 3: Neutral (Some effort to differentiate, but with confusion)
   - 4: Agree (Clear differentiation, but some minor overlap)
   - 5: Strongly Agree (Clear and consistent differentiation)
