# Supplementary material for: Evaluation of Primary Care Behavioral Health (PCBH) with guided self-help CBT as a treatment option – a protocol of a single-blind randomized multicenter trial (KAIROS)
Source: BMC Health Serv Res. 2025 Sep 23;25:1208. doi: 10.1186/s12913-025-13232-4 (PMC12455819; doi:10.1186/s12913-025-13232-4)
Supplement: Supplementary file 6 — Supplementary Material 6 [file 12913_2025_13232_MOESM6_ESM.docx]

Supplementary materials F

Details on the decision-support algorithm and its use in the retrospective suitability assessment

# Details on the retrospective assessment

The algorithm combines questionnaire scores with patient-rated vignettes describing different issues. Patients rate how well each vignette matches their experience (0–4) and how important it is to address (1–4). These ratings are multiplied, generating scores from 0–16 per problem area. The algorithm further adjusts scores by assigning 15 points to the problem area the patient ranks as most important and 10 points to the second most important. Additionally, if a patient scores above the cut-off on the relevant questionnaire, indicating the problem area may be significant, 10 extra points are added for that area. These adjustments result in a total score ranging from 0 to 51. This score is used in the retrospective assessment as detailed in Figure S1.

Fig. S1. Flow chart of the retrospective assessment algorithm for assessing indication for structured treatment.


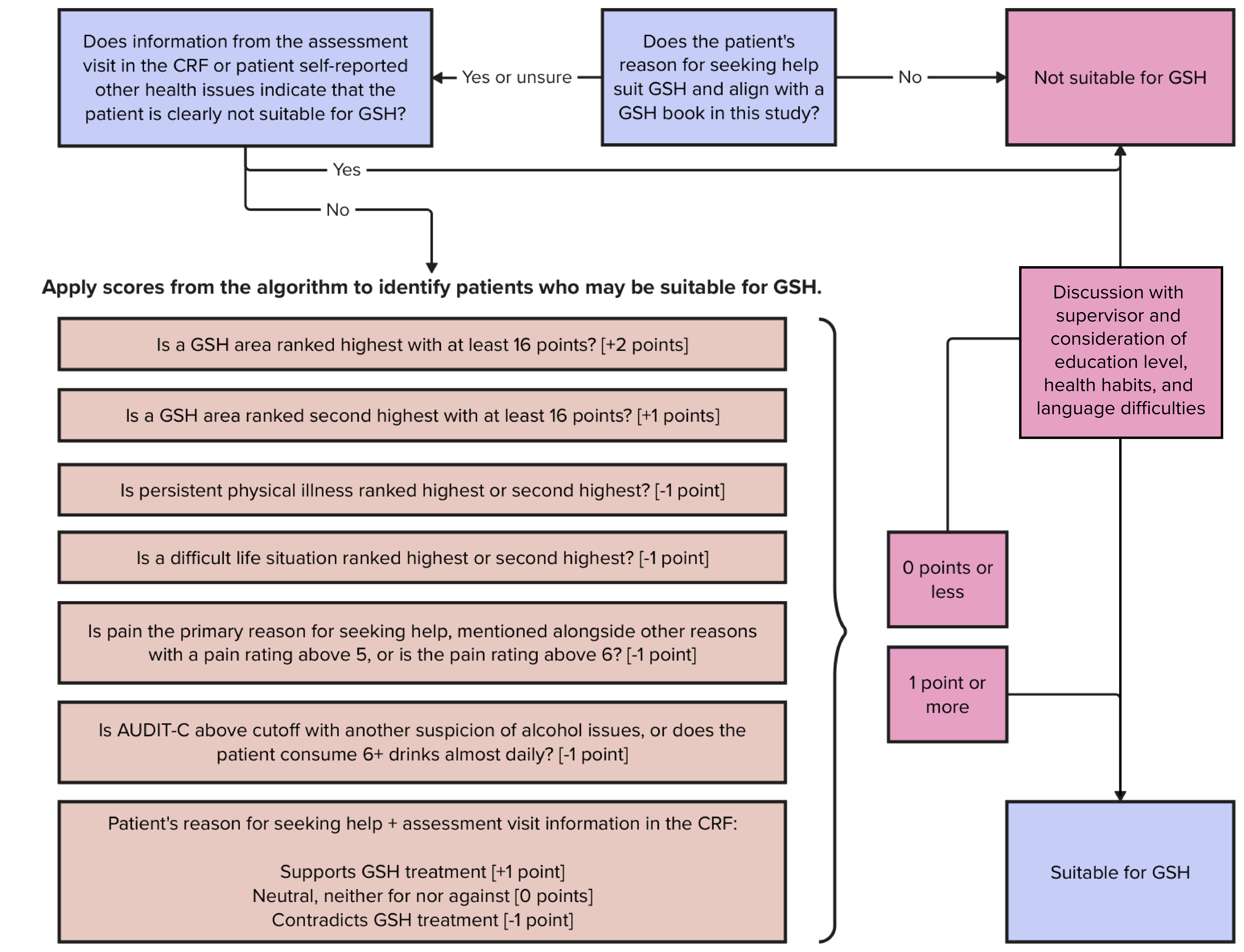


# Vignettes

The following vignettes, translated from Swedish, were used in the algorithm.

## Depression

“Most of us feel down from time to time. Sometimes, this feeling becomes deeper and more persistent, often referred to as depression. The reasons for feeling down or depressed vary widely and manifest differently from person to person. Life changes, such as losing significant people or activities that once brought joy, can trigger sadness. It is also common to feel down due to other factors like sleep problems, anxiety, pain, or difficult life circumstances.

When experiencing depression, it is typical to lose interest in activities that once brought pleasure. Things that used to feel enjoyable may no longer evoke positive emotions. Repetitive thoughts and rumination about problems can become overwhelming. Self-doubt, guilt, and negative thoughts about life are common. Thoughts about death or life’s meaninglessness may also arise. Despite increased thinking, clarity diminishes, and attention and energy levels decline. Decision-making becomes difficult, and motivation for tasks that need to be done decreases. Feeling physically drained and becoming passive are also common experiences.”

## Worry and anxiety

“It is normal to feel anxious before important events or when facing challenges. Anxiety helps us anticipate and prepare for different scenarios. However, for some, anxiety takes up too much space in daily life. Constant worry, often disproportionate to the actual situation, can revolve around work, family, health, or fears of something bad happening. Worries may shift depending on life circumstances. Instead of helping us cope, anxiety becomes a persistent discomfort and limitation. It can feel difficult to control and drains both time and energy. Relationships may suffer, especially if frequent reassurance is sought from loved ones. Despite being aware of excessive worrying, stopping can be challenging.

Feeling down or overly sensitive is common when life is perceived as problematic or threatening. Stress may persist even when actual demands are manageable. Anxiety can also lead to sleep problems and physical symptoms such as muscle tension and pain.”

## Panic attacks and panic disorder

“A panic attack involves a sudden and intense experience of physical and emotional discomfort. The anxiety response is similar whether triggered by a specific fear, such as spiders or social situations, but panic disorder refers to when these feelings arise unexpectedly and without a clear cause. Panic disorder also includes a fear of the physical sensations themselves, often leading to concerns about dying, fainting, or losing control. Sometimes, the fear of having another panic attack can itself trigger one.

Typical physical symptoms include a rapid heartbeat, difficulty breathing, chest pressure, dizziness, and trembling, along with other unpleasant bodily sensations. During a panic attack, there is an overwhelming sense of fear and distress.

Many who have experienced panic attacks begin avoiding places or situations associated with them. This often includes public transportation, enclosed spaces, or being alone. Some develop safety behaviors such as carrying water, taking medication, or ensuring they are always accompanied. These avoidance behaviors can significantly impact daily life and, in some cases, severely limit what individuals feel capable of doing.”

## Social anxiety

“For those with social anxiety, being the center of attention can cause profound discomfort. There is often a fear of embarrassing oneself or being scrutinized by others. It is common to feel as if all eyes are on you in social settings, making it difficult to stay present.

When experiencing this discomfort, the body reacts. Blushing, sweating, dry mouth, dizziness, and difficulty concentrating are typical responses. Stuttering and trembling may occur, and some fear specific reactions such as shaking hands, blushing, or experiencing a mental block.

To manage social situations, individuals may develop coping strategies. They might attend gatherings but find them highly stressful. Many with social anxiety start limiting themselves in both personal and professional settings due to overwhelming discomfort, despite a genuine desire for more involvement. This restriction can lead to feelings of sadness or frustration.”

## Stress-related problems

“Stress arises when something is perceived as a potential threat, activating the fight-or-flight response. Attention sharpens, and the body prepares for action. While short-term stress can be beneficial, prolonged intense stress can negatively impact well-being. Common triggers include juggling too many responsibilities or feeling overwhelmed by demands at home or work. Stressful situations where there is a lack of control, insufficient time, or an unstructured routine also contribute.

It is common to fall into negative cycles, prioritizing tasks over self-care (such as leisure, relationships, rest, and sleep) in an effort to stay productive. This can lead to physical and mental exhaustion. Persistent muscle tension, headaches, and stomach aches are common symptoms, and concentration and decision-making may become difficult. If stress persists for too long without recovery, severe symptoms can develop, potentially leading to complete exhaustion.”

## Sleep problems

“Sleep problems involve persistent difficulty sleeping over an extended period. Sometimes, the cause is clear, such as stress, being woken by children, or working shifts. However, sleep issues can also persist without an obvious reason, making it essential to address them directly.

Common sleep problems include insufficient sleep or misaligned sleep patterns. Difficulties may involve taking a long time to fall asleep, waking frequently, or waking too early.

Prolonged sleep disturbances often impair daily functioning. Concentration becomes challenging, irritability increases, and emotional detachment may occur. Sleep and mental well-being influence each other: mental health issues often worsen sleep quality, while inadequate sleep can further impact overall well-being.”

## Illness anxiety

“Occasionally worrying about health is normal. However, illness anxiety, or hypochondria, involves excessive fear of having or developing a serious illness, such as cancer or heart disease. This fear persists over time and significantly impacts quality of life. Anxiety may be triggered by reading about diseases or noticing bodily sensations. Symptoms associated with the feared illness are often experienced.

Many with illness anxiety seek medical attention more frequently than others, while some avoid medical facilities altogether, even when care is needed. Relationships with family members may be strained, especially if reassurance is frequently sought. Long-term illness anxiety can lead to frustration and sadness. If these fears recur frequently, they can interfere with daily activities and increase passivity.”

## Obsessive compulsive disorder

“OCD consists of both obsessions and compulsions.

Obsessions are recurring, intrusive thoughts that cause significant distress. Common themes include fear of contamination, harming others, or catastrophic events. These thoughts often lead to compulsions—actions performed to reduce distress.

Some compulsions are directly linked to obsessions, such as excessive washing due to contamination fears or repeatedly checking appliances to prevent fires. Others, like excessive arranging, may not have an obvious connection. Compulsions can also include mental rituals aimed at suppressing distressing thoughts.

OCD can cause significant distress, as individuals may spend large portions of their day caught in cycles of obsessions and compulsions. Many begin avoiding triggers, which can greatly limit daily life.”

## Persistent physical illness

## “Chronic physical conditions include diabetes, asthma, cardiovascular diseases, gastrointestinal disorders like ulcerative colitis, and neurological conditions such as MS and Parkinson’s. Chronic pain conditions, tinnitus, and irritable bowel syndrome (IBS) also fall into this category.

## Living with a long-term condition can profoundly impact daily life. It often leads to increased reliance on others, reduced work capacity, and social isolation. Meaningful activities may become more challenging, affecting overall quality of life. Managing symptoms often takes precedence over enjoying life’s positive aspects.”

## A difficult life situation

## “People sometimes seek help when life becomes overwhelming. Stressors may include caregiving responsibilities without respite, financial difficulties, loneliness, or relationship dissatisfaction. Workplace problems, such as excessive workload or conflicts, can also be contributing factors. Threats or violence in close relationships or having a loved one with substance abuse issues are other common stressors. Often, multiple challenges combine, making the situation feel unmanageable.”

# References

1. Hallgren M, Kraepelien M, Öjehagen A, Lindefors N, Zeebari Z, Kaldo V, et al. Physical exercise and internet-based cognitive-behavioural therapy in the treatment of depression: randomised controlled trial. Br J Psychiatry J Ment Sci. 2015 Sep;207(3):227–34.

2. Kraepelien M, Blom K, Lindefors N, Johansson R, Kaldo V. The effects of component‐specific treatment compliance in individually tailored internet‐based treatment. Clin Psychol Psychother. 2019;26(3):298–308.
