# Supplementary material for: Evaluation of Primary Care Behavioral Health (PCBH) with guided self-help CBT as a treatment option – a protocol of a single-blind randomized multicenter trial (KAIROS)
Source: BMC Health Serv Res. 2025 Sep 23;25:1208. doi: 10.1186/s12913-025-13232-4 (PMC12455819; doi:10.1186/s12913-025-13232-4)
Supplement: Supplementary file 8 — Supplementary Material 8 [file 12913_2025_13232_MOESM8_ESM.docx]

Supplementary materials H

All items from the World Health Organization Trial Registration Data Set

| **Data category** | **Information** |
| --- | --- |
| Primary registry and trial identifying number | ClinicalTrials.gov: [NCT05335382](https://clinicaltrials.gov/show/NCT05335382) |
| Date of registration in primary registry | May 25^th^, 2021 |
| Secondary identifying numbers | 2020-04198 |
| Source(s) of monetary or material support | Kamprad Family Foundation for Entrepreneurship, Research and Charity (2019-2024), Karolinska Institutet Funds (2020-2021), Capio Närsjukvård (2019-2027), the Swedish Research Council (2021-06481), Regional agreement on medical training and clinical research (ALF) between Karolinska Institutet and Region Stockholm (FoUI-977722). |
| Primary sponsor | Linnaeus University |
| Secondary sponsor(s) | Karolinska Institutet |
| Contact for public queries | *AFVC* anneli.farnsworthvoncederwald@lnu.se |
| Contact for scientific queries | *AFVC* Department of Psychology, Faculty of Health and Life Sciences, Linnaeus University, Växjö, Sweden. |
| Public title | The KAIROS Project |
| Scientific title | Evaluation of Primary Care Behavioral Health (PCBH) with guided self-help CBT as a treatment option – a protocol of a single-blind randomized multicenter trial (KAIROS) |
| Countries of recruitment | Sweden |
| Health condition(s) or problem(s) studied | All mental and behavioral health issues commonly presenting in primary care |
| Intervention(s) | Experimental Arm:  Extended Primary Care Behavioral Health (PCBH) with guided self-help as a treatment option for those suitable Active Comparator Arm: Core PCBH with only brief interventions as a treatment option |
| Key inclusion and exclusion criteria | Inclusion criteria: adult patient (≥ 18 years) who seeks primary care at participating unit, deemed to be suitable for behavioral health interventions Exclusion criteria: does not speak Swedish well enough to fill out questionnaires, is deemed to need emergency type care (such as immediate risk of suicide) |
| Study type | Interventional Allocation: 1:1 individual randomization Intervention model: parallel assignment Masking: single-blinded Primary purpose: treatment |
| Date of first enrolment | June 14th, 2021 |
| Target sample size | 983 |
| Recruitment status | Recruiting |
| Primary outcome(s) | Daily functioning |
| Key secondary outcomes | Costs, care process factors, symptoms, satisfaction, subjective change, adverse events, treatment content, medication prescription, sick leave, care consumption |
