# Supplementary material for: Evaluation of Primary Care Behavioral Health (PCBH) with guided self-help CBT as a treatment option – a protocol of a single-blind randomized multicenter trial (KAIROS)
Source: BMC Health Serv Res. 2025 Sep 23;25:1208. doi: 10.1186/s12913-025-13232-4 (PMC12455819; doi:10.1186/s12913-025-13232-4)
Supplement: Supplementary file 9 — Supplementary Material 9 [file 12913_2025_13232_MOESM9_ESM.docx]

Supplementary materials I

Model consent form

# Information for research participants

We would like to ask if you would be willing to participate in the KAIROS research project. This document provides information about the KAIROS project and what participation entails.

## What is this project, and why are you asking me to participate?

Linnaeus University and Karolinska Institutet, in collaboration with [the primary care center where you are seeking care], are conducting the KAIROS project to improve the quality of psychosocial support and treatment in primary care. We aim to assess whether closer collaboration between different professional groups and increased access to psychological treatment can better support patients with mental and behavioral health issues. The study will evaluate both the effectiveness of this approach and compare two different types of extended patient assessments.

We are inviting you to participate in the study because the healthcare professionals you have been in contact with believe you may benefit from a visit with a psychologist, counselor, or therapist. Please read this information carefully. If you decide to participate in the study, you will provide your consent further down in this document.

## How does the study work?

Participants in the study will be randomly assigned to one of two types of extended assessment, both of which are more comprehensive than the standard assessments typically conducted in primary care:

1. **An extended assessment** focusing on your mental well-being, daily functioning, and the type of support you feel you need. Before your first visit, you will answer questions online or on paper, which takes 15–30 minutes. Your responses will help both you and your clinician determine the best course of care.
2. **Same as (1), but with an additional structured interview** about your mental health.

After four, eight, and twelve weeks, as well as after one year, you will be asked to answer additional questions online or on paper about your well-being, any changes you have made, and your experience of the care received. We may also contact you by phone to gather complementary information or to ask about your experience with the care provided. Long-term follow-ups of up to three years may be conducted, and we will contact you if this becomes relevant. Your medical records will be documented in the usual manner. Your participation in the research study will not negatively affect your access to other healthcare services.

We will also collect data on sick leave, medication use, and healthcare consumption from the following public registers:

- The **Prescribed Drug Register**,
- The **Swedish Patient Register**,
- **MIDAS** (Swedish Social Insurance Agency),
- **Primary Care Quality** (Swedish Association of Local Authorities and Regions), and
- One of the regional registers **VAL** (Region Stockholm), **SHR** (Region Skåne), or **VEGA** (Region Västra Götaland), if you reside in one of these regions.

## What are the risks?

The extended assessments and subsequent interventions have been previously tested in research and routine care without any identified risks. However, answering questionnaires and interviews may be perceived as demanding, taking between 10 and 30 minutes per session. Working on changes that you and your clinician agree upon may also require time and can be emotionally challenging, similar to psychological treatment in general.

## Are there any benefits?

The purpose of evaluating treatments provided in primary care is to improve the quality of psychosocial support and treatment. In the long run, we hope these improvements will benefit all primary care patients. The immediate benefit of participating in the study is that the initial questions you answer may help both you and your clinician gain a better understanding of your situation and needs, leading to more informed treatment decisions. It is currently unknown whether adding a structured interview to the assessment improves outcomes compared to self-report questionnaires and self-reflection, and this is what we aim to investigate.

## What happens to my data?

The KAIROS project will collect and record information about you based on:

- The questions you answer throughout the study,
- Assessments made by healthcare professionals,
- Your medical records, and
- Data from public registers.

To conduct a scientific evaluation of each treatment, we will link this data to your personal identification number. Data will be stored digitally in a secure database, with access controlled by the responsible researcher. Paper records will be stored according to the primary care center’s patient data management routines and later entered into the database.

Once all study data has been collected, it will be extracted from the secure database in a **pseudonymized format**, meaning each participant is identified only by a code. This data will be stored on a secure server at Linnaeus University. The code key will remain in the secure database so that individuals cannot be identified during data analysis. The results will be statistically compiled and published in scientific journals and conferences without any individual responses being traceable.

Healthcare professionals at your primary care center involved in your treatment will have access to the questionnaire responses you provide but **will not** have access to data from public registers. They may choose to use this information as a tool in determining your care, but it is up to them to decide whether to review your responses. **Therefore, you should not use your responses in the KAIROS project as a way to communicate information to your healthcare provider—any important details should be shared with them directly.**

All information will be handled confidentially, and standard patient confidentiality rules apply. The data will be analyzed by researchers at Linnaeus University and Karolinska Institutet. Data will be stored for **your lifetime** to enable future research.

The **data controller** responsible for your personal information is Linnaeus University. Personal data is processed under the **EU General Data Protection Regulation (GDPR)** for research purposes, based on the legal basis of public interest. Under GDPR, you have the right to:

- Access your personal data,
- Request corrections if needed,
- Request the deletion of your data,
- Restrict the processing of your data.

If you have questions or wish to access your data, contact Linnaeus University’s Data Protection Officer at **0470-76 75 78** or **dataskyddsombud@lnu.se**. If you are dissatisfied with how your data is handled, you can file a complaint with the Swedish **Data Protection Authority** at **datainspektionen@datainspektionen.se** or **08-657 61 00**.

## How will I be informed about the study results?

The results of the KAIROS project will be published in **Open Access** scientific journals, making them freely available if you wish to read them when they are published.

## Insurance and compensation

This study is conducted within **regular healthcare services**, meaning there is no financial compensation or exemption from healthcare costs. **Standard patient insurance applies.**

## Participation is voluntary

Your participation is **voluntary**, and you may withdraw at any time. If you choose not to participate or decide to withdraw, you do not need to provide a reason, and this will **not** affect your future care or treatment. If you wish to withdraw, please contact the KAIROS project’s contact person. You may also inform a member of the research team during an interview or other direct interaction.

## Contact person for questions about the KAIROS project and your participation

**Anneli Farnsworth von Cederwald**
Department of Psychology, Linnaeus University
351 95 Växjö, Sweden
Phone: **072 237 23 00**
Email: **anneli.farnsworthvoncederwald@lnu.se**

## Research sponsor and data controller

The **research sponsor** (the organization responsible for the study) and **data controller** is **Linnaeus University**.

## Responsible researcher

**Viktor Kaldo, Professor and Licensed Psychologist**
Linnaeus University and Karolinska Institutet
Email: **viktor.kaldo@lnu.se**

## Consent to participate in the study

I have received both verbal and written information about the study and have had the opportunity to ask questions. I can keep/print/copy this written information.

☐ I consent to participate in the KAIROS project.
☐ I consent to my personal data being processed as described in the study information.

**Mobile number:** _________________________________________
**Place and date:** _________________________________________
**Signature:** ______________________________________________
